# Water Harvesting by Thermoresponsive Ionic Liquids: A Molecular Dynamics Study of the Water Absorption Kinetics and of the Role of Nanostructuring

**DOI:** 10.1021/acs.jpcb.3c01655

**Published:** 2023-06-02

**Authors:** Nancy C. Forero-Martinez, Robinson Cortes-Huerto, Lainey Ward, Pietro Ballone

**Affiliations:** † Institut für Physik, Johannes Gutenberg-Universität Mainz, Staudingerweg 9, 55128 Mainz, Germany; ‡ Max-Planck Institute for Polymer Research, Ackermannweg 10, 55128 Mainz, Germany; § School of Physics, University College Dublin, UCD Belfield Campus, D04V1W8 Dublin 4, Ireland; ∥ Conway Institute for Biomolecular and Biomedical Research, University College Dublin, UCD Belfield Campus, D04V1W8 Dublin 4, Ireland

## Abstract

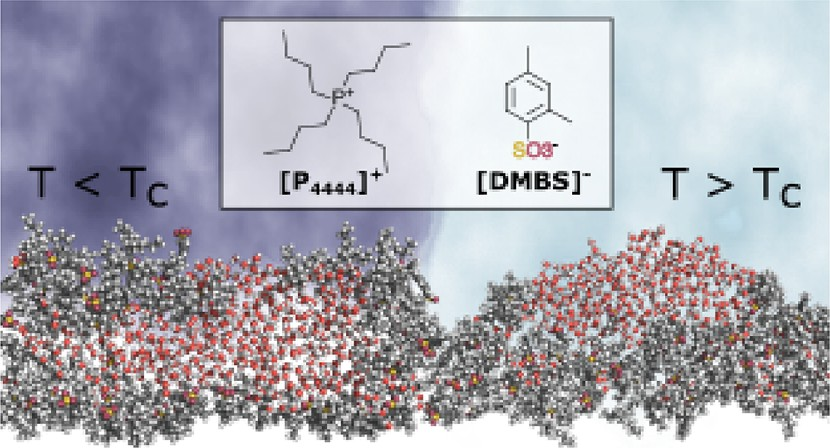

Ionic liquids (ILs) whose water solutions are thermoresponsive provide an appealing route to harvest water from the atmosphere at an energy cost that can be accessed by solar heating. IL/water solutions that present a lower critical solution temperature (LCST), i.e., demix upon increasing temperature, represent the most promising choice for this task since they could absorb vapor during the night when its saturation is highest and release liquid water during the day. The kinetics of water absorption at the surface and the role of nanostructuring in this process have been investigated by atomistic molecular dynamics simulations for the ionic liquid tetrabutyl phosphonium 2,4-dimethylbenzenesulfonate whose LCST in water occurs at *T*_c_ = 36 °C for solutions of 50–50 wt % composition. The simulation results show that water molecules are readily adsorbed on the IL and migrate along the surface to form thick three-dimensional islands. On a slightly longer time scale, ions crawl on these islands, covering water and recreating the original surface whose free energy is particularly low. At a high deposition rate, this mechanism allows the fast incorporation of large amounts of water, producing subsurface water pockets that eventually merge into the populations of water-rich and IL-rich domains in the nanostructured bulk. Simulation results suggest that strong nanostructuring could ease the separation of water and water-contaminated IL phases even before macroscopic demixing.

## I Introduction

Securing fresh water for the ever-expanding needs of humankind in a time of climate change is a crucial challenge for our society.^1,2^ With traditional resources already exploited to the limit and often overexploited, innovative sources are expected to play an increasing role in the future. For instance, desalination of seawater and other brackish water reservoirs already accounts for an estimated 10^8^ tons of fresh water a day, covering about 1% of global consumption.^3^ Vast landlocked areas, however, lack even low-quality water suitable for desalination and still require increasing amounts of fresh water to maintain their population and agricultural productivity. The marginal resource available virtually everywhere is a nonvanishing concentration of vapor in the atmosphere, which accounts for 0.04% of the fresh water on our planet.^4,5^ Where climatic conditions are favorable, water vapor condenses into dew or fog, whose harvesting has been exploited for irrigation since ancient times^6^ and, at present, is still used in advanced agricultural practice.^7^

In vast arid and semiarid regions, however, vapor density and temperature never reach the condensation point, negating the minimal conditions for economical water harvesting. Even in these cases, however, the vapor concentration generally exceeds 10 g/m^3^, and water molecules can be extracted from the atmosphere, using a variety of approaches and materials that have been proposed to this aim and, to a limited extent, already deployed for applications.^8^ A nonexhaustive list includes thermodynamic traps (collectors)^9^ as well as composite solid sorbents,^10^ based on complex materials such as activated carbon fibers,^11^ silica gels,^12^ zeolites,^13^ and metal organic frameworks (MOFs).^14−16^ The simplest approaches are based on the idea of absorbing undersaturated water vapor into a suitable hygroscopic material.^17−19^ Besides being limited in the achievable water concentration, spontaneous absorption is necessarily driven by a decrease of free energy in going from the vapor to the absorbed state, making the eventual production of fresh water even more unlikely or more energy-demanding than in the starting vapor state.

The initial loss of free energy, however, might turn into an advantage if the absorbing system is responsive to some external parameter, whose variation triggers the change of the phase equilibrium of the water-sorbent combination, favoring the separation of water.^20^ In this context, thermoresponsive polymer–water mixtures have already been considered and partly developed, reaching the initial testing stage.^21^ Selected organic ionic liquids (ILs) whose water solutions are thermoresponsive might also play a crucial role^22^ and could offer some advantages with respect to polymer-based materials. First, their optimal composition for thermoresponsiveness often requires less water than for polymers. Then, thermoresponsive IL/water solutions also display nanostructuring, whose nature and potential role in water harvesting are discussed below. In systems of this kind, water and ionic liquids cross a mixing-demixing transition upon a modest temperature change, limited to about 30 °C, comparable to the day-night temperature variation in several semiarid regions. Since the water vapor saturation is highest at the low night temperature, and water might be more needed during the hot daylight hours, the best choice to implement this water harvesting strategy is an IL whose water mixture presents a lower critical solution temperature (LCST), being mixed at low *T* and demixing with increasing *T*. If this absorption, separation, and IL-regeneration cycle could be achieved using the natural day-night temperature oscillation, a new promising approach to water harvesting could be devised even for arid environments, requiring almost only inexpensive energy from the environment. The specific water production per day of this approach is expected to be similar to that of methods based on silica gels or MOFs, i.e., of the order of the kg/day/m^2^,^23^ corresponding to the absorption of a homogeneous water film about 1 mm thick per day.

While the thermodynamics of the water harvesting cycle can be determined accurately and virtually completely based on existing data, minimal information is available on kinetic aspects that might indeed decide the feasibility of water harvesting through ILs. More traditional solid sorbents, for instance, are porous and present a vast specific surface area (up to 1380 m^2^/g in the case of activated carbon fibers).^24^ Therefore, it is important to assess whether the faster dynamics of a liquid surface or any other structural and dynamical property may compensate for the reduced area. Other aspects, whose role in water harvesting is equally uncertain, might also be relevant. For instance, the known cases of IL/water thermoresponsive solutions present a second-order demixing transition around room temperature at nearly 50–50 wt % IL-water composition. Away from this equal composition, the mixing-demixing transition becomes weakly first-order and moves to higher temperatures beyond the day-night temperature oscillation range. In practice, at the 1 mm/day absorption rate, water concentration in IL as high as 50 wt % might not be attainable. However, virtually all known cases of IL/water thermoresponsiveness are accompanied by the nanostructuring of the nominally homogeneous solution, consisting of the formation of IL-rich and water-rich domains and occurring over a broad IL-water relative concentration range. Moreover, again on the nominally homogeneous side of the phase diagram, the size of the IL-rich and water-rich domains increases with temperature approaching the demixing line, becoming macroscopic at the transition. If nanostructuring is sufficiently strong, i.e., if the water domains in an IL-rich system are big enough and sufficiently different in composition and properties, then macroscopic separation could be achieved using simple mechanical methods, provided the system remains fluid at all temperatures and concentrations of interest. For instance, one could rely on the sizable difference in density (up to 50%) of the two components to achieve separation of the IL-rich and water-rich phases by gravity, possibly enhanced by mild centrifugation.

Again, simulations and experiments show that, in both nanostructured and phase-separated conditions, the water-rich phase is fairly pure.^25,26^ However, it might need to be further purified to make it suitable for drinking and agriculture and, perhaps even more importantly, to fully recover the IL. This stage might require filtration by reverse osmosis, although again under very mild conditions, since the osmotic pressure of the water-rich phase is already low. The IL-rich phase typically contains up to 10% water, whose presence, however, might have a positive effect on the harvesting process since it eases the kinetics of water absorption, nanostructuring/phase separation, nanodomain equilibration, and migration through the sample.

To contribute to the overall design and initial development of the harvesting approach, by atomistic molecular dynamics (MD) simulations we investigate: (i) the kinetics of water absorption through the surface of an IL whose water solutions are known to demix at an LCST point; (ii) the nanostructuring at the surface of the corresponding IL/water solutions on the miscible side of the mixing/demixing line. In other words, we investigate how the inhomogeneous and anisotropic conditions of a free surface affect nanostructuring and demixing in IL/water solutions.

Our simulations concern water solutions of tetrabutyl phosphonium ([P_4444_]^+^) 2,4-dimethylbenzenesulfonate ([DMBS]^−^), whose thermoresponsiveness and nanostructuring in the bulk have been investigated by experiments and simulations in ref (25). The experimental LCST occurs at *T*_c_ = 36°*C* and *x*_c_ ≈ 50–50 wt % composition. The structural aspects of interest for our interfacial study can be investigated by considering relatively small systems. However, the high viscosity of ILs forces us to cover relatively long time scales up to the μs.

The simulation results show that, from a chemical-physics point of view, water harvesting using thermoresponsive ILs is likely to be feasible. In particular, water sequestration below the [P_4444_][DMBS] surface occurs spontaneously. Up to high water concentration it requires microscopic times (∼10^–7^ s) and gives origin to sizable subsurface water-rich domains. The successive evolution toward an equilibrium population of domains spread over the system volume is much slower. However, the kinetics of water domains migrating to deeper layers, making absorption a volume and not a surface process, covers time scales much longer than those accessible by atomistic MD. The effectiveness of this process could be enhanced, for instance, by increasing the minimum amount of water to be left in the IL-rich phase after water extraction. Moreover, mechanical stirring could also help to achieve bulk water uptake.

## II Model and Method

**Figure 1:**
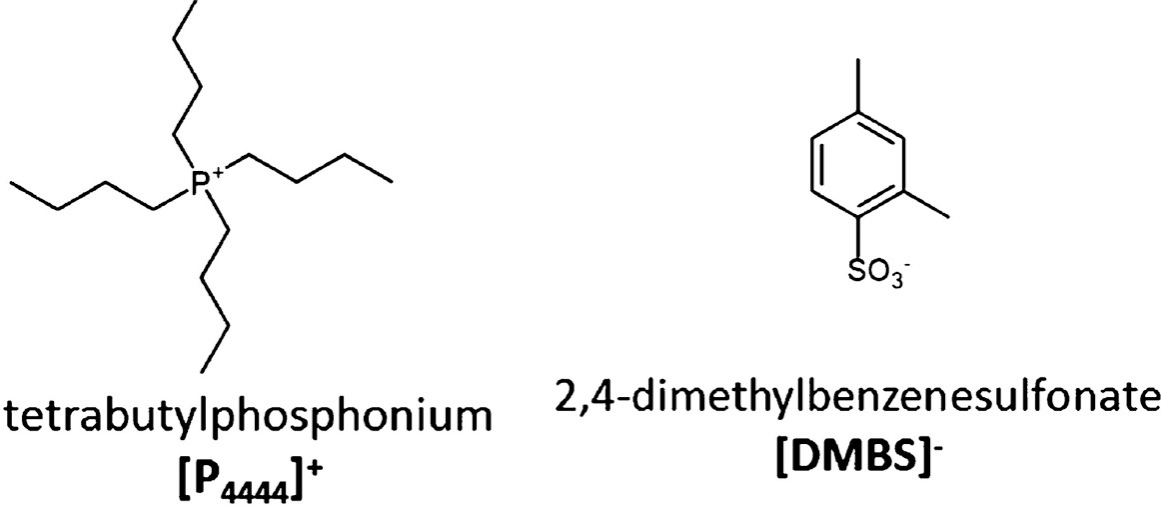
Schematic structure of the ions considered in the present study.

Among the different IL compounds whose water solutions are thermoresponsive, in our simulations we considered [P_4444_][DMBS], whose schematic structure is shown in Figure 1 and whose nanostructuring and phase diagram in the bulk have already been investigated and discussed in detail in ref (25).

In the present study, the force field for [P_4444_][DMBS] is of the Gromos (version 54a7) type,^27^ whose parameters have been obtained through the ATB web page.^28^ The SPC water model^29^ has been used in the development of Gromos 54a7 to tune the absorption properties of organic molecules. For the sake of consistency, we used the same SPC model in the simulations. All simulations are carried out using the Gromacs package^30^ version 2019.

To validate this model, preliminary investigations of structural and thermodynamic properties of nominally homogeneous (possibly nanostructured) phases were carried out by MD simulations in the NPT ensemble using cubic samples and periodic boundary conditions. To investigate the water absorption kinetics in the IL, a slab geometry was adopted and simulated under NVT conditions. In all samples, the IL component is represented by 1728 neutral ion pairs, while the amount of water ranges from zero to 13 220 molecules, corresponding to a weight composition going up to 25 wt % water concentration. This range is significantly below the optimal composition range of thermoresponsiveness for [P_4444_][DMBS]/water solutions, which is close to 50–50 IL/water wt % concentration. The reason for our choice is as follows. On the basis of the data for MOFs sorbents, one can expect an absorption rate of ∼1 mm water/day, corresponding to a slightly sub-millimeter IL layer to achieve 50 wt % composition. The difficulty of dealing with, collecting, and processing such a thin liquid-like layer over large areas might force the usage of somewhat thicker layers, in which the water concentration after a single loading stage will be less than 50 wt %. Moreover, because of the high viscosity of pure ILs, any kinetic problem affecting absorption and water mobility will be more apparent the lower the water content, making the low water concentration samples the most relevant for our investigations.

The slab geometry is enforced by enclosing the sample into a periodically repeated orthorhombic simulation box of sides {*L*_*x*_, *L*_*p*_, and *L*_*p*_}, where *L*_*x*_ > *L*_*p*_ and the *p* label of *L*_*p*_ refers to the *yz* plane, and all interactions are computed considering periodic boundary conditions (pbc). The box volume has been chosen significantly larger than the equilibrium volume of the simulated systems; therefore, the samples will be inhomogeneous, comprising both a liquid (possibly glassy at the lowest *T* and lowest water concentration) and a vapor phase, consisting of only a few water molecules and no IL. Because of the nonvanishing surface tension of all samples and since *L*_*x*_ > *L*_*p*_, the system will form a slab whose fluctuating planar surfaces are orthogonal to the *x* axis, in such a way to minimize the surface area.

**Figure 2:**
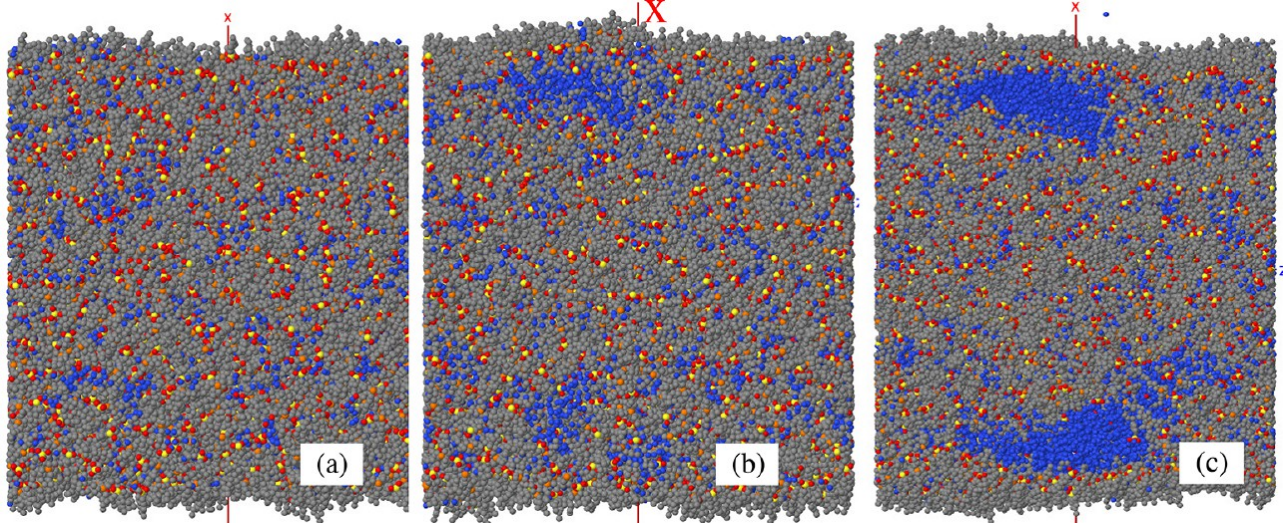
Simulation snapshots of representative samples at *T* = 300 K. All samples consist of a planar liquid slab orthogonal to the *x* axis, made of 1728 [P_4444_]^+^ [DMBS]^−^ ion pairs and 4320 water molecules, on which have been deposited (sum of the two sides): (a) zero water molecules; (b) 2800 water molecules (about 3/4 ML); (c) 8900 water molecules (about 2.5 ML). Gray dots: carbon in the ions; red dots: oxygen in the ions; blue dots: water oxygen; yellow dots: S in [DMBS]^−^; orange dots: P in [P_4444_]^+^. Hydrogen is not shown.

Slab simulations have been carried out in the NVT ensemble in which the constant temperature is enforced through a Nosé–Hoover thermostat whose parameters correspond to a relaxation time of 2 ps for every kinetic energy fluctuation. The system is being simulated at 11 equispaced temperatures spanning the range 260 ≤ *T* ≤ 360 K (*T* = 260, 270, ..., 360 K), thus covering the experimental *T*_c_ = 36 °C of the equicomposition solution. In reality, water crystallizes at *T* = 273 K and nearly boils at *T* = 360 K, but the mixing with [P_4444_][DMBS] extends the stability range of the liquid solution. The size of the sample and its basic structure can be appreciated in the snapshot from simulations at *T* = 300 K shown in Figure 2. Because of the two parallel free surfaces and of the fluid or nearly fluid state of the sample that (well inside the slab) makes the virial tensor isotropic, the pressure in the sample is negligible, corresponding to the vanishingly small vapor pressure of the ionic liquid for the pure IL samples or to the moderate vapor pressure of water for the [P_4444_][DMBS]/water solutions.

To summarize, the simulated samples consist of [P_4444_][DMBS]/water solutions with up to 170 × 10^3^ atoms, enclosed in cubic (homogeneous or nanostructured three-dimensional (3D) samples) or orthorhombic (slabs) cells, periodically repeated in space. The moderate size allows for long simulation times, extending up to the μs time scale, but also implies that the properties of the slabs are largely dominated by surface effects, which, however, are also the major targets of our study.

The analysis of trajectories includes the visual inspection of snapshots, the plot of the average potential energy as a function of temperature, the determination of water and ion profiles across the slab, the determination of diffusion coefficients along the surface to characterize the liquid or glassy state of the samples, and mobility orthogonal to the surface to assess the ability of species to redistribute themselves across the system.

The self-diffusion coefficient, in particular, is computed through the Einstein relation based on the asymptotic slope of the mean square displacement for species σ as a function of time. To account for the anisotropy of the slab samples, one first computes
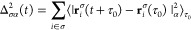
1where the average is performed with respect to τ_0_, and the asymptotic limit of interest is that for long times. Moreover, α labels Cartesian coordinates. In the slab case, we will distinguish Δ_*x*_^2^ and Δ_∥_^2^ = Δ_*y*_^2^ + Δ_*z*_^2^ to characterize mobility and diffusion along the directions orthogonal and parallel to the surface, respectively. In the homogeneous, isotropic case, we will drop the subscripts and consider Δ^2^ = Δ_*x*_^2^ + Δ_*y*_^2^ + Δ_*z*_^2^, from which a unique self-diffusion coefficient is determined.

The dilute vapor filling the space in between periodically repeated slabs might spoil the computation of the water diffusion constant, because of the high mobility of water in the vapor phase. To overcome this problem, in a very empirical way, in eq 1 we discard all contributions |**r**_*i*_^σ^(*t* + τ_0_) – **r**_*i*_^σ^(τ_0_)|^2^ such that either *x*_*i*_^σ^(*t* + τ_0_), *x*_*i*_^σ^(τ_0_) or both are outside the slab, with the limits of the slab determined a posteriori for each system from the computed atom number density profile, being conventionally located at 10% of the bulk value. We verified that, in all cases, the uncertainty in the computed diffusion constant for water is relatively minor, apparently because the interslab volume is small, and up to high *T* the vapor pressure of water is low. This problem with diffusion does not concern the ions, because their high cohesive energy prevents their evaporation from the slab.

The nanostructuring of water in the samples is investigated in real space, by dividing the water molecules into disjoint clusters of geometrically connected molecules and determining the statistical distribution of their sizes. To this aim, we use a homemade software, which determines all *n*_*p*_-connected groups of molecules, where the basic connectivity between two water molecules requires a OW-OW distance of less than 3.2 Å, where OW is the water oxygen. Then, *n*_*p*_-connectivity means that each molecule in the cluster is connected at least *n*_*p*_ times to water molecules in the same cluster. At low water concentration, we will focus primarily on the simple 1-connectivity. With an increasing number of water molecules in the sample, approaching the percolation limit, simple connectivity loses its ability to identify nanostructuring, and we will resort to *n*_*p*_ > 1 connectivities, the most relevant case being *p* = 2. In this procedure, no request is made on the orientation of water molecules. Therefore, the connectivity defined in this way is not based on the network of water–water hydrogen bonds, although water molecules whose OW atoms are separated by less than 3.2 Å are very likely to be H-bonded.

The most relevant thermodynamic property of surfaces and interfaces is the surface tension γ, representing the free energy cost of creating a new surface of unit area.^31^ Free energies are difficult to determine by simulation because of their entropy contribution but nevertheless are computed using thermodynamic and mechanical (variation of the tangential pressure) approaches. We found it convenient to estimate the surface tension by monitoring the equilibrium fluctuations of the liquid/vapor interface, which increase the instantaneous surface area and, for this reason, are controlled by the surface tension. The method to carry out the computation of γ is described in detail and discussed in Section I of the Supporting Information document.

Auxiliary simulations concerned homogeneous samples, carried out under NPT conditions, imposed using the Parrinello–Rahman barostat. In this case, diffusion properties are represented by the usual 3D isotropic diffusion coefficient, and nanostructuring is characterized both by the real-space clustering described in the previous paragraph and by suitable structure factors. This second analysis starts by computing the Fourier transform of three species, i.e., ions (species 1, 2) and water (species 3) with
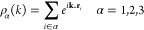
2Then, partial structure factors are computed according to
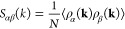
3where *N* is the total number of ions for (α, β) = (1, 1), (1, 2), (2, 2), and the number of water molecules for (3,3) (see ref (32)). The average indicated by ⟨···⟩ is over configurations along the trajectory and reciprocal lattice vectors **k** such that |**k**| = *k*. In our analysis, ions and water molecules are coarse grained, being represented by a single particle located at the P atom position for [P_4444_]^+^, at the S atom position for [DMBS]^−^, and at the OW position for water. Water-rich domains are identified through prepeaks in the *S*_3,3_(*k*) at *k* < 1 Å ^–1^. Nanostructuring of [P_4444_][DMBS] and its water solutions is identified through a similar prepeak in the density–density structure factor of the ionic compound, defined as
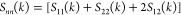
4The low-*k* range of the complementary charge–charge structure factor
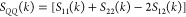
5reflects the perfect screening of the Coulomb charges, and it does not show any anomaly even in case of nanostructuring. In the case of IL/water solutions, the low-*k* peak due to nanostructuring in the *S*_3,3_(*k*) and in *S*_*nn*_(*k*) are related, but the one in *S*_3,3_(*k*) is more apparent at relatively low water concentration (water-in-salt conditions), while the one in *S*_*nn*_(*k*) is more apparent at relatively low IL concentration (salt-in-water conditions).^33^

The thermoresponsive demixing as well as its preliminary manifestation as nanostructuring are greatly affected by the temperature dependence of the number and quality of hydrogen bonds connecting water molecules to anions and among themselves. The close relation depends on the fact that the entropy term that drives the mesoscopic or macroscopic separation of IL and water is due predominantly (but not exclusively) to the different entropy cost/advantage of forming/breaking hydrogen bonds among water molecules or between water and anions. In atomistic models based on empirical force fields, the only possible definition is in terms of the geometry of OW-H–O triplets, where the second oxygen atom may belong to another water molecule or to [DMBS]^−^. In our analysis, the triplet forms a hydrogen bond if the distance between the two oxygen atoms is less than 3.2 Å and the OW-H–O angle is wider than 140 degrees.

Although unusual in a *Method* section, we mention here what we did not use, i.e., free energy sampling methods,^34−36^ that, in principle, could be used to speed up the system equilibration and to compute free energy profiles for all species across the slab, thus providing unambiguous insight on water absorption free of simulation time limitations.^37^ However, the very viscous character of [P_4444_][DMBS] and the nanostructured state of its water solutions make this analysis very uncertain or exceedingly time-consuming.

## III Results

### A Dry and Water-Contaminated [P_4444_][DMBS] Homogeneous Samples

A preliminary set of simulations has been carried out to characterize the structural and thermodynamic properties of pure and water-contaminated [P_4444_][DMBS] samples, in either the homogeneous or slab geometry. All samples contain 1728 neutral pairs of [P_4444_]^+^, [DMBS]^−^ ions.

As already stated, 11 temperatures have been considered, from *T* = 260 K to *T* = 360 K, covered in steps of 10 K. The results shown in this section concern primarily simulations at relatively high temperature (*T* ≥ 300 K), whose equilibration is the fastest to achieve and the easiest to verify, but the full interval of temperatures has been simulated for each sample. As discussed below, no change of phase is observed in the simulated systems; therefore, the results presented for the highest temperatures summarize the properties also at lower temperature, although the kinetics of the different processes might be significantly different at *T* < 300 K and *T* ≥ 300 K.

Simulations of homogeneous dry [P_4444_][DMBS] samples, lasting 120 ns at each temperature, have been carried out with the aim of: (i) verifying whether the system is strictly homogeneous or it is nanostructured, due to formation of charged and neutral nanodomains; (ii) computing thermodynamic properties and the diffusion coefficients to assess the liquid or glassy state of the samples.

The results show that the average potential energy *U*(*T*) depends almost linearly on temperature (see Figure S1 in Supporting Information), with only a slight curvature that requires a careful numerical analysis to be quantified. In other words, as expected, there is no sign of discontinuity due to full or partial crystallization. Less obviously, there is also no rapid change in the slope of *U*(*T*) that could point to a clear glass transition at a *T*_g_ in the 260 ≤ *T*, K ≤ 360 range.

At all temperatures, the computation of the mean square displacement Δ^2^(*t*) as a function of time shows some residual mobility and a wide linear range of Δ^2^(*t*) versus *t*. Over the explored temperature range, the diffusion coefficients of the ions change from 1 × 10^–9^ cm^2^/s at the lowest *T* to 1 × 10^–7^ cm^2^/s at the highest *T*. At *T* = 300 K, in particular, the diffusion coefficient is *D*_+_ = (2.1 ± 0.3) × 10^–8^. Despite the mass advantage, anions diffuse at virtually the same rate, having *D*_–_ = (1.9 ± 0.3) × 10^–8^ cm^2^/s. The diffusion data are not sufficiently accurate (especially at *T* < 300 K) to allow an Arrhenius analysis of the results, but overall, the analysis of mobility confirms that the system is indeed fluid, although very viscous. Last but not least, the experimental *T*_g_ of a series of similar IL made of tetrabutylphosphonium cations and amino acid anions^38^ are close to 200 K, supporting the fluid state of pure [P_4444_][DMBS] over the simulated temperature range. Needless to say, the liquid state of the system at all temperatures of interest is crucial for any application in water harvesting.

**Figure 3:**
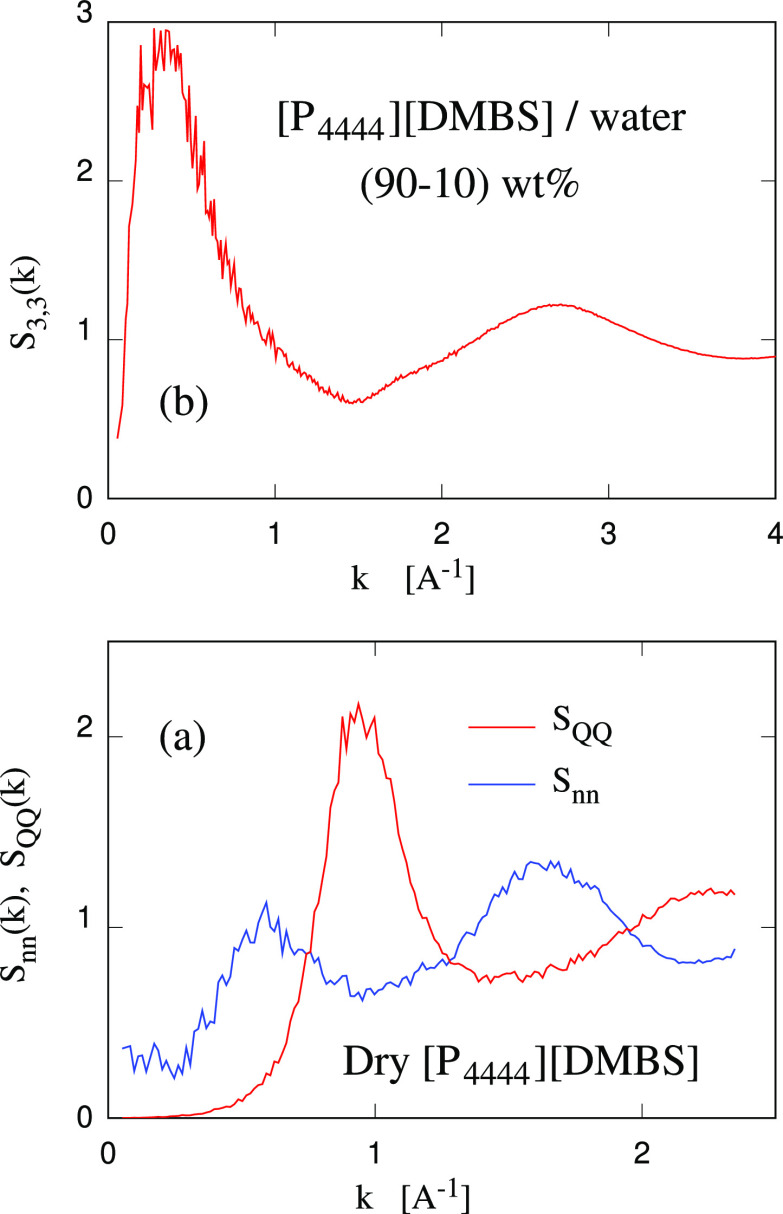
(a) Density-density (*S*_*nn*_) and charge–charge (*S*_*QQ*_) structure factors of a 3D extended [P_4444_][DMBS] sample at *T* = 300 K. While the simulation is atomistic, the analysis is coarse-grained, with each ion represented by a single particle located at the *P* and *S* position of [P_4444_]^+^ and [DMBS]^−^, respectively. (b) Water–water structure factor *S*_3,3_(*k*) for the [P_4444_][DMBS]/water solution at (90–10) wt % concentration. Data were from the *T* = 290 K, *P* = 1 bar simulation.

At all temperatures, the density–density structure factor *S*_*nn*_(*k*) has a prepeak at *k* = 0.55 Å ^–1^, corresponding to a real space periodicity of ∼11 Å, longer than the real space periodicity of ∼4 Å corresponding to the main peak of *S*_*nn*_(*k*) at *k* ≈ 1.6 Å ^–1^. We interpret the prepeak as due to nanostructuring, although its strength and length scales appear to be moderate (see Figure 3). We emphasize that this nanostructuring is not the one discussed here and in other papers^25^ for [P_4444_][DMBS]/water solution. In the pure IL, nanostructuring is related to the local aggregation of the neutral moieties of both [P_4444_]^+^ and [DMBS]^−^, as discussed, for instance, in ref (39) and in the review paper ref (40).

The concern about the system being in a glassy state is much decreased upon adding 10 wt % of water to the sample. The choice of this composition for our preliminary investigation of the homogeneous phase is due to the fact that experimental studies^25^ as well as independent simulations for [P_4444_][DMBS]/water at 50–50 wt % concentration show that, upon phase separation at *T* > *T*_c_, the IL-rich phase contains ∼10 wt % water. For this reason, the (90–10) wt % IL/water solution is considered here as the baseline system, used for absorbing water and suitable to be easily regenerated at the end of the water harvesting cycle. Since this sample and composition will be mentioned several times in what follows, for the sake of simplicity we will refer to it as the (90–10) wt % sample or slab, without a more specific characterization. Simulations show a clear favorable effect of water on the system kinetics, with a temperature-dependent but always sizable increase in the diffusion coefficient of the ions with respect to the dry sample case. At *T* = 300 K, for instance, the diffusion coefficients of the ions in the wet slab are *D*_+_ ≈ (3.7 ± 0.3) × 10^–8^ cm^2^/s and *D*_–_ = (2.6 ± 0.3) × 10^–8^ cm^2^/s. Moreover, in the (90–10) wt % sample, the diffusion coefficient of water (*D* = (3.65 ± 0.1) × 10^–6^ cm^2^/s at *T* = 300 K) is 2 orders of magnitude higher than that of the ions, suggesting that already at this composition single water molecules are able to reach relatively quickly an equilibrium distribution. The diffusion properties of ions and water in the equilibrium samples are illustrated in Figure S2 and Figure S3 of Supporting Information. It is apparent that the fluidizing effect of water is stronger on the cation that on the anion. This feature is likely to be due to the formation of multiple hydrogen bonds between water and each anion, increasing its effective mass and introducing a sticky interaction that hampers the [DMBS]^−^ mobility.

**Figure 4:**
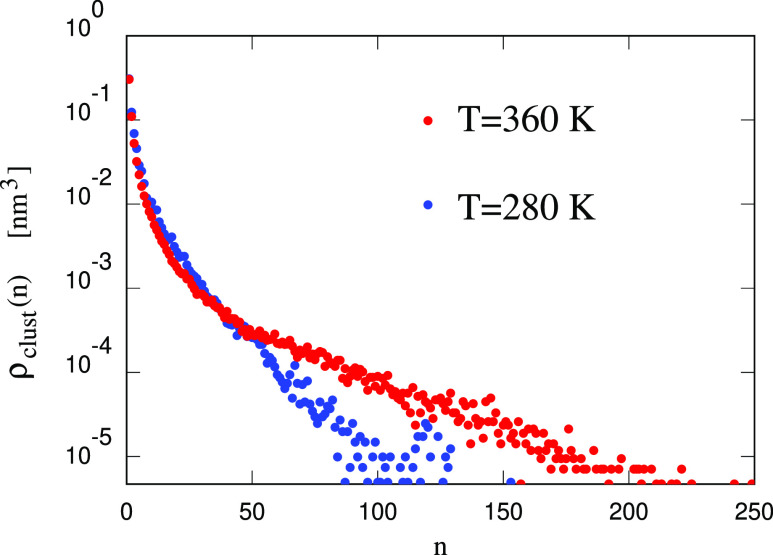
Density distribution (on a logarithmic scale) of water clusters of size *n* (measured in number of water molecules) in the slightly hydrated (10 wt % water) 3D extended sample. Blue dots: *T* = 280 K; Red dots: *T* = 360 K.

Computation of the *S*_3,3_(*k*) (water–water) partial structure factor shows that the distribution of water molecules in the (90–10) wt % sample is nanostructured, as apparent from the peak at *q* ≈ 0.45 Å ^–1^ (see Figure 3b). Although powerful for homogeneous systems, the *S*_3,3_(*k*) analysis of the water distribution is less suitable for inhomogeneous systems with surfaces and interfaces. To cover both homogeneous and inhomogeneous cases, we reanalyzed the clustering of water molecules using the real space method described in Section II. In this homogeneous case, the result of the real space analysis is equivalent to that offered by the *S*_*αβ*_(*k*)’s. Nanostructuring is apparent, and the size distribution of (1-connected) water clusters extends to higher sizes with increasing *T* (see Figure 4), consistent with the fact that IL and water nanostructuring becomes stronger at high *T*, anticipating a hypothetical phase separation that at this low water density could occur at *T* > 100 °C. At all temperatures, visualization of the largest clusters in the (90–10) wt % sample reveals connected but rather open structures (see Figure S4 in Supporting Information), certainly not suitable to be separated by simple mechanical means from the IL environment.

Nanostructuring and its increase with increasing *T* are reflected in the number and quality of hydrogen bonds (HBs) in the (90–10) wt % sample. From the molecular structure of the ions, it is easy to see that the addition of water to [P_4444_][DMBS] will introduce H-bonding between water and anions in addition to water–water HBs. Water will act simultaneously as the donor and acceptor, while [DMBS]^−^ will exclusively accept HBs. Despite the wt % imbalance, the water and [DMBS]^−^ components have a comparable ability to accept HBs. At *T* = 300 K, on average, each of the 4320 water molecules in the sample donates 0.70 HBs to [DMBS]^−^, implying that each of the 1728 [DMBS]^−^ anions will accept 1.75 HBs from water. Moreover, at the same *T* = 300 K, each water molecule donated (and receives) on average 0.80 HBs to (from) water. With increasing *T*, the total number of HBs decreases relatively slowly, but the decrease in the number of water–anion HBs is significantly faster than the decrease of water–water HBs (See Figure S5 in Supporting Information), pointing to a shift in the relative stability of the two types of HBs, reflecting the tendency toward increasing separation of the two components with increasing *T*. Nevertheless, the decrease in water-[DMBS]^−^ HBs is continuous at all temperatures, without evidence of macroscopic demixing at this low water concentration.

### B Dry and Water-Contaminated [P_4444_][DMBS] Slabs

The homogeneous samples of the previous simulations, always with 1728 neutral ion pairs, were turned into planar slabs by extending the side *L*_*x*_ of the simulation box by ∼40%. The transformation from homogeneous sample to slab has been discontinuous, having been accomplished by changing the *L*_*x*_ value in the input and using Gromacs run time options to reconstruct molecules split by pbc, relocating them into the central box. After equilibrating during ∼30 ns, production runs lasted from 120 to 150 ns at each temperature.

**Figure 5:**
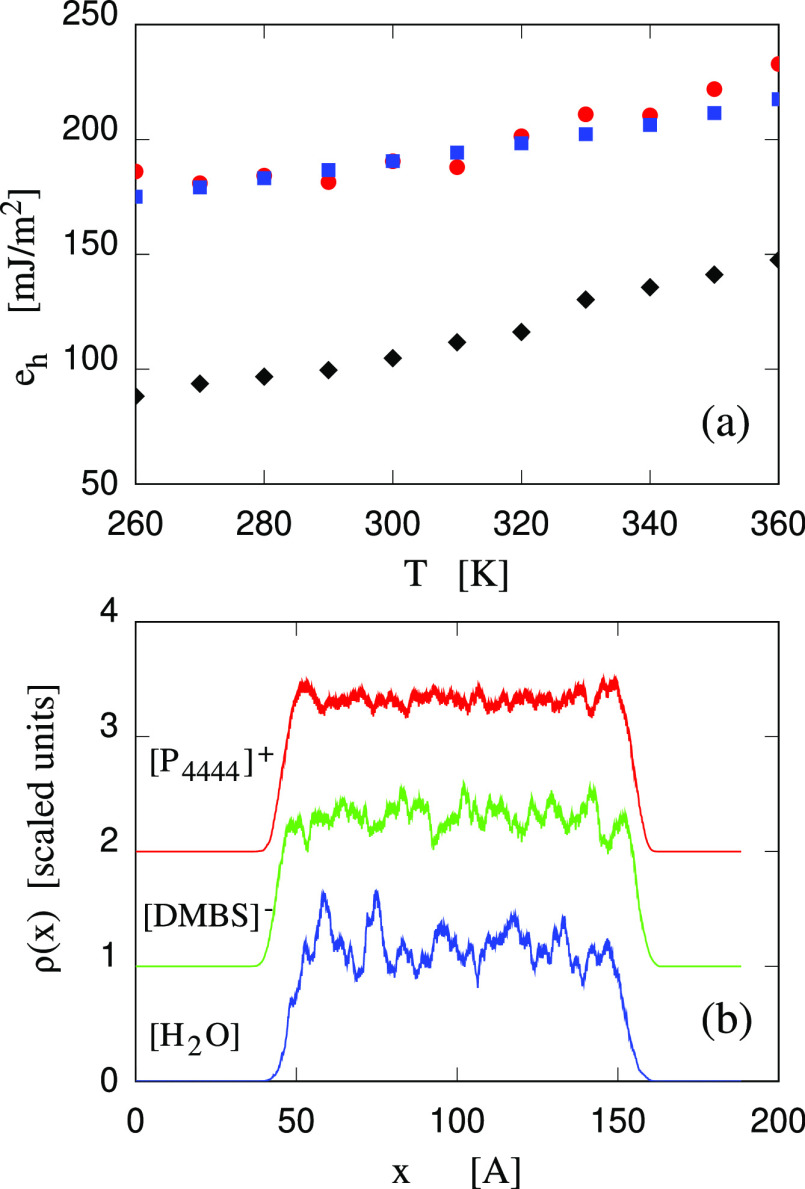
(a) Surface energy as a function of temperature. Black diamonds: [P_4444_][DMBS]; red dots: [P_4444_][DMBS]/water at (90–10) wt % concentration; blue squares: pure water. (b) Density profile across the slab of the ions and water. Sample consisting of [P_4444_][DMBS]/water at (90–10) wt % concentration; *T* = 300 K. The three profiles have been scaled differently to bring them on the same range of values and shifted along the *y* axis for the sake of clarity.

Despite the high cohesive energy of [P_4444_][DMBS], the surface energy of the dry sample is around 100 mJ/m^2^, i.e., roughly half of that of water (Figure 5a). The surface energy grows faster than linearly with increasing *T*, pointing to a gain of entropy through the enhanced molecular disorder at the surface, possibly affecting primarily the neutral tails of both ions, as discussed in the next paragraph.

The density profiles of cations and anions across the slab show relatively sharp interfaces, whose 90%–10% width (∼1 nm) is only slightly larger than the size of the ions. The same density profiles show little layering of atoms parallel to the surface; the charge density is low nearly everywhere and without much structuring, apart from short wavelength contributions of relatively low amplitude that very likely represent long-lived thermal fluctuations. This observation does not imply that cations and anions are kinetically bound to each other but only that, in the absence of clear layering, the planar average of the charge density virtually vanishes. The most relevant structural feature of the surface, apparent also from snapshots, is that both ions at the surface orient their butyl ([P_4444_]^+^) and aryl groups toward the vacuum, explaining the low surface energy.

Mobility of the ions along the surface (two-dimensional (2D)) is low but not negligible, consistent with the 3D diffusion in the homogeneous samples. On time scales of the order of 100 ns, the mean square displacement of both ions in the *x*-direction and along the surface are comparable, implying that mobility along *x* is slightly faster, at least on the stated time scale, than along *yz*. On significantly longer time scales, the finite width of the slab will certainly reverse the result of the comparison.

More interesting in view of applications are the results for the surface of the sample with 10 wt % water. From the point of view of the ions, the structure of the surface is nearly unchanged with respect to the dry case. Interestingly, but also predictably, water stays just inside the outermost ionic layer (see Figure 5b), leaving again the low-energy hydrocarbon tails exposed to the surface (see Figure S6 in Supporting Information). Also in this case, the surface energy is computed by comparison with the homogeneous sample of an equal average water content. Somewhat surprisingly, given the similarity of the topmost layer composition, the surface energy nearly doubles with respect to the dry case, bringing the result close to that of water (Figure 5a). A possible explanation is that water molecules link anions through hydrogen bonds, enhancing the 3D connectivity of the system. Cutting this network below the surface significantly contributes to the surface energy. This feature, however, deserves further investigation since the near coincidence of the solution surface energy with that of water is very intriguing, given the fact that virtually no water is in the topmost layer of the sample. The interpretation of the high surface energy in terms of the binding effect of water on [P_4444_][DMBS] is supported by the computation of the mixing enthalpy of [P_4444_][DMBS] and water at 10 wt % composition, showing a sizable negative value (see Table 1), and, even more importantly, by the fact that this composition corresponds to the IL-rich phase resulting from the LCST transition, making it a well-defined phase in itself.

**Table 1:** Mixing Enthalpy kJ/mol per Water Molecule for the [P_4444_][DMBS]/Water System at (90–10) wt% Composition

*T* [K]	260	270	280	290	300	310	320	330	340	350	360
*h*_mix_	–4.9	–5.2	–5.8	–6.0	–6.3	–6.1	–5.9	–5.2	–4.5	–3.8	–2.6

Besides the surface energy, however, the most important thermodynamic property of surfaces and interfaces is the surface tension^31^ γ, whose computation is challenging because it requires the estimation of entropy or, more precisely, of the entropy difference between the system with the surface and the corresponding extended system. In our study, the surface tension of the liquid surfaces has been estimated through the equilibrium fluctuations of the surface around its planar average position, using the method outlined in Section I of the Supporting Information document. The characterization of the fluctuations in the surface position of the dry, (90–10) wt % and pure water slabs is illustrated and briefly discussed in Figure S7 of Supporting Information. Because of the high viscosity of the dry and (90–10) wt % samples, we are unable to reliably average the amplitude of the fluctuations of longest wavelength compatible with our pbc conditions, making the quantitative analysis somewhat uncertain, but still able to show trends and semiquantitative values. The fitting of |*C*_q_|^2^ by (*k*_B_*T*)/[*A*(*γq*^2^ + *k*_c_*q*^4^)] shows that, at *T* = 300 K, γ is the same to within the (sizable) error bar in the dry and (90–10) wt % slabs, in both cases being about one-third of the water surface tension. Considering also the data for the surface energy, this result confirms that a crucial factor for the low surface tension of [P_4444_][DMBS] and of its water solutions is the surface entropy of the nonpolar hydrocarbon moieties of both ions. In turn, the high surface entropy and correspondingly low surface tension are the forces driving the fast incorporation of water below the surface of the solutions.

The clustering of water molecules in the (90–10) wt % slab is similar to that of homogeneous samples, although slightly but systematically enhanced by confinement. Also the number of HBs in the system and their separation into the water–anion and water–water varieties are nearly the same as in the bulk sample, being again only slightly enhanced by confinement. The water density profile across the slab does not show any sign of this nanostructuring, since averaging over the *yz* plane removes any evidence of clustering.

The mobility of water along the surface is consistent with the 3D diffusion measured in the homogeneous sample, meaning that evaluated at equal time
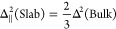
6Mobility along the direction *x* orthogonal to the surface is not associated with diffusion, since the square displacement Δ_*x*_^2^ is bound from above. Nevertheless, already within 100–150 ns, Δ_*x*_^2^ acquires large (temperature-dependent) values of the order of magnitude of the square width of the slab, showing that water molecules can travel across the slab fairly easily and within microscopic time scales.

### C Water Absorption on the Dry and Water Contaminated [P_4444_][DMBS] Slab

The kinetics of water absorption at the surface of [P_4444_][DMBS] has been investigated starting from the dry and the water-contaminated [(90–10) wt %] slabs described in the previous section. To this aim, water molecules have been added at random positions in the interlayer space, separating periodically repeated slabs. Water addition has been carried out in batches of 1000 water molecules, briefly relaxing the sample for 1–3 ns between successive additions, up to a preset number of added water molecules. Following the preparation stage, long equilibration and production runs have been carried out at constant composition, lasting at least 150 ns but reaching up to and exceeding the μs for selected samples, as specified below.

For all compositions and at all temperatures, water molecules from the vapor impinge on an outermost liquid layer consisting almost exclusively of the hydrocarbon moieties of both [P_4444_]^+^ and [DMBS]^−^ ions. Despite the predominantly hydrophobic character of this surface, water molecules stick to the surface, becoming physisorbed, within a microscopic time of the order of the ns. Perhaps precisely because of the hydrophobic layer, water molecules do not immediately enter the slab but migrate on the surface forming 3D islands. Only at the highest temperatures (*T* > 320 K), a small but noncompletely vanishing population of water molecules is observed in the vapor phase.

Then again, in all cases and at all temperatures, on a still microscopic time scale water finds itself into a subsurface position, covered by cations and anions reforming a hydrophobic outermost layer. As discussed in the previous section, the reason for this preference is the low surface tension of [P_4444_][DMBS] compared to that of water. This stage of subsurface incorporation or, more precisely (as discussed below), of ions moving outward and covering the water molecules, takes place on time scales of the order of 100 ns. The further evolution of the sample toward a mixed, nanostructured, or phase-separated equilibrium state might take up to macroscopic time scales. Despite the considerable length of a few of our simulations and the limited thickness of the slabs, such an equilibrium state might not have been reached in at least a few cases. Nevertheless, the results on the μs time scale provide useful indications on the feasibility of water harvesting using thermoresponsive ILs.

#### 1 Thousand Water Molecules Deposited on the Dry [P_4444_][DMBS] Slab

More in detail, at all explored temperatures, adding 1000 water molecules to the dry IL slab of 13.5 × 13.5 nm^2^ cross section (water weight fraction of ∼2%), placing them in the empty interslab space, quickly results in their adsorption on the surface, due primarily to dispersion interactions between the water oxygen and the hydrocarbon tails of the ions. Considering the volume of the water molecule (*V*_*w*0_ = 0.030 65 nm^3^, assumed to be a cube) at normal conditions, this number of molecules on the two surfaces corresponds to a coverage of slightly more than 1/4 of monolayer (ML) per surface. In the confined geometry of the surface, clustering of molecules is enhanced, giving origin to 3D water islands, whose formation and morphology also confirm the hydrophobicity of the outermost layer.

**Figure 6:**
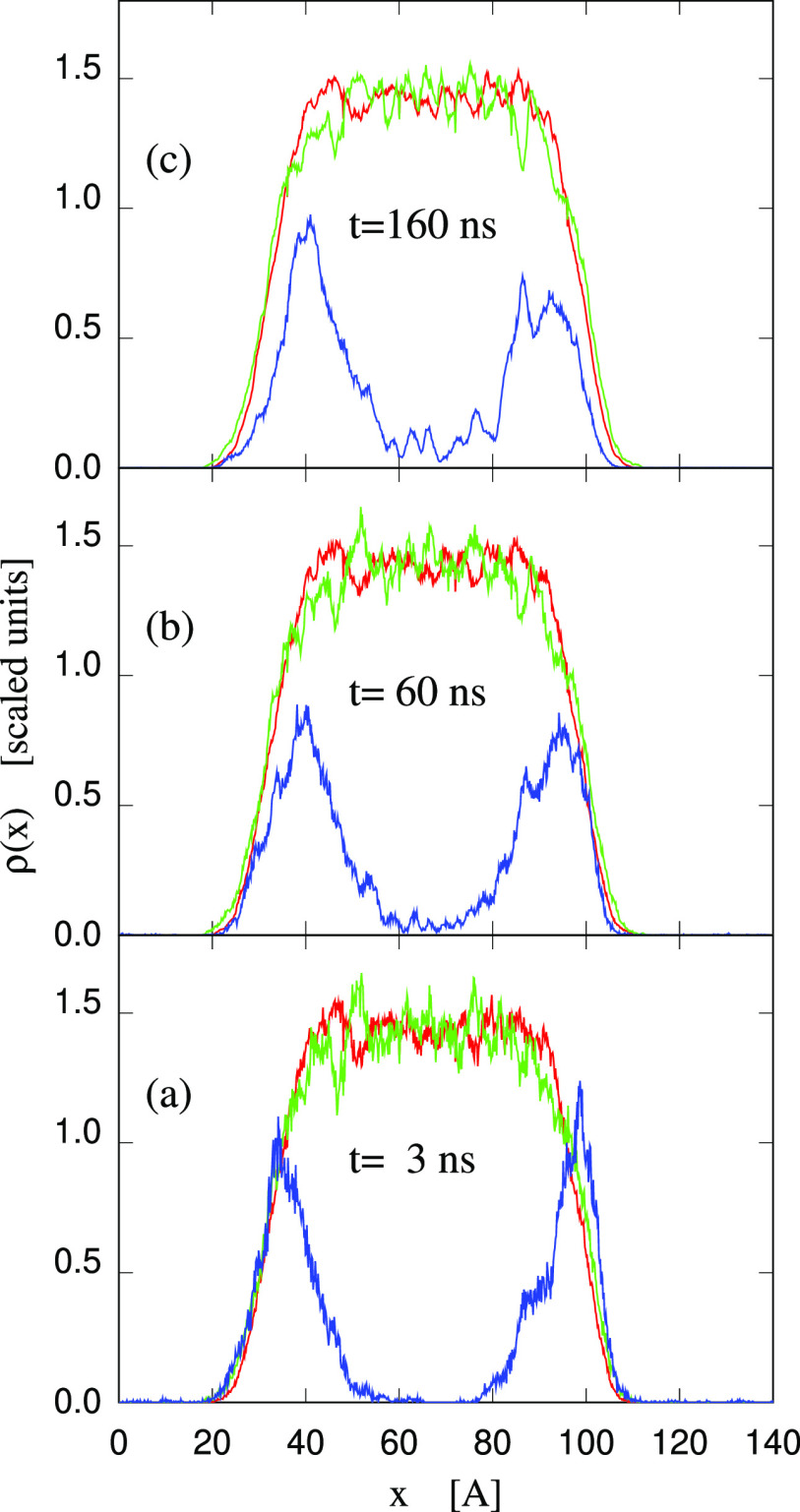
Time evolution of the ion and water density profile following random addition of 1000 water molecules in the interlayer space between periodically repeated dry IL slabs. The simulated sample consisted of 1728 [P_4444_][DMBS] ion pairs and 1000 water molecules. Red line: [P_4444_]^+^; green line: [DMBS]^−^; blue line: H_2_O. Virtually all water molecules are physisorbed or absorbed below the IL surface within 3 ns (a) and fully buried below the surface within 100 ns (b). Further progression toward the inside of the slab is much slower (c). Density units have been scaled differently for the three components to bring them on the same range of values.

This overlayer structure, however, is unstable or weakly metastable, since water quickly finds its way toward subsurface positions. To be precise, animations of the simulation trajectories suggests that, at least for water islands consisting of >100 molecules, the water incorporation does not occur through molecule-by-molecule diffusion of H_2_O into the salt. Instead, we observe whole water islands being progressively covered by ions that crawl on their surface, keeping their Coulombic moiety directed toward water. In this configuration, the hydrocarbon tails of ions isolate the charged and polar groups of ions and water molecules from the vacuum side of the interface, represented by the ∼40 Å gap between periodically repeated slabs. Beyond this stage, which lasts about 100 ns, the evolution of the water distribution along *x* is very slow, as shown again in Figure 6 summarizing the simulation results for the *T* = 300 K slab.

On the basis of several considerations, we think that, at this low water concentration, the broad subsurface peaks of Figure 6 are likely to be only a transient feature and that, eventually, the equilibrium water distribution will resemble that of Figure 5b, whose overall water content is comparable. Judging from the simulation data, it is possible to estimate that, at *T* ≤ 300 K, full equilibration and the spreading of the ions over the simulated samples will take place on the order of a few μs. This picture is supported by the results of simulations carried out at *T* > 300 K. For instance, at the highest simulated temperature, i.e., *T* = 360 K, the two subsurface peaks dissolve relatively quickly, with the water density profile becoming similar to the unstructured one in Figure 5b.

At all temperatures, however, even though the density distribution along *x* might become flat on average, the 3D distribution of water molecules is not homogeneous at the single molecule or ion scale but rather nanostructured, as shown by the analysis of clustering. At these low concentrations, however, clusters are relatively small (up to 40–50 water molecules); their structure is open and not likely to significantly ease the separation of water and IL. In many ways, these clusters represent a good example of water in salt nanostructuring.^33^

Nevertheless, the burying of the water islands below the surface, taking place within ∼100 ns even at low temperature, is an important aspect of the system evolution, implying that the surface will remain unaltered during water absorption, always presenting the same hydrophobic top layer with an ionic, hydrophilic layer immediately below. It might be worth emphasizing again that the process of incorporating water subsurface is not based on single water molecules diffusing inside, as observed in other cases,^37^ but is a collective effect, consisting of ions crawling on the surface of water islands and covering them. This, in turn, suggests that the subsurface incorporation is irreversible since the reverse process of ions moving away and exposing patches of water prone to evaporation appears to be highly unlikely. All of these aspects are confirmed by the results of the simulations with increasing water coverage discussed below.

#### 2 One ML Water Deposited on the (90–10) wt % Slab

Admittedly, the absorption of water into the dry sample is not the focal point of our study, since the dry IL is an idealization, both because of the hygroscopic character of ILs and because of the phase diagram, showing that the IL-rich phase that can easily be regenerated through thermoresponsiveness contains about 10 wt % of water. Therefore, we simulated more extensively the absorption of water on the (90–10) wt % IL-water sample, starting from a sample made of the (90–10) wt % slab, to which 2800 water molecules have been added, corresponding to nearly 1 ML adsorption on each exposed face of the slab.

**Figure 7:**
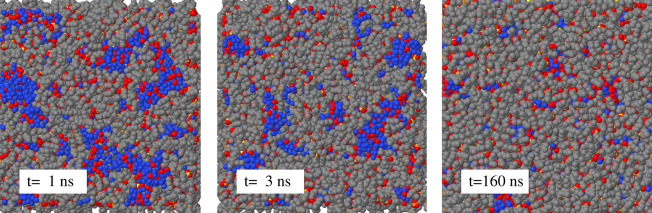
Time evolution of the surface of the (90–10) wt % slab with 2800 additional water molecules deposited on the two surfaces. Gray dots: carbon; red dots: oxygen in [DMBS]^−^; yellow dots (hardly visible): P in [P_4444_]^+^; blue dots: O in water.

The results for this simulation turn out to be similar to those of the previous case with some differences due to the higher water concentration in the whole sample. A pictorial summary of the sample evolution at *T* = 300 K is shown in Figure 7. At first, water molecules form islands on the [P_4444_][DMBS] surface within 1 ns. Islands are not 2D but have a thickness comparable to the lateral size. The presence of each island bends the IL surface inward, forming concavities in which the island settles. Both the discrete island formation and their thickness confirm again the hydrophobic interaction. Within a few more ns, ions start to diffuse over the island’s surface, eventually covering water with a monolayer film of ions. As before, this process takes of the order of 100 ns. Since this process is more easily visualized for larger and thicker islands, it will be analyzed in more detail below, when presenting the data for higher water content. The mobility of water molecules is sizable, both parallel and orthogonal to the surface, and there is a relatively fast mixing of water molecules already in the slab and those newly added. To be precise, in a first stage mixing is due primarily to water molecules already in the (90–10) wt % slab, moving toward the surface and diffusing into the adsorbed water islands. The reverse process, due to isolated adsorbed molecules diffusing inside the (90–10) wt % slab, occurs only rarely. The density profile of cations, anions, and water reflects this evolution. As in the previous case, on the 200 ns time frame, the water distribution presents two prominent subsurface peaks, located at about 15 Å below the top atomic layer, consisting again primarily of hydrocarbon tails. After the first ∼50 ns, at *T* = 300 K the evolution of the subsurface peaks becomes very slow, suggesting that, despite the good mobility of water molecules, water clusters do not dissolve into the IL substrate.

**Figure 8:**
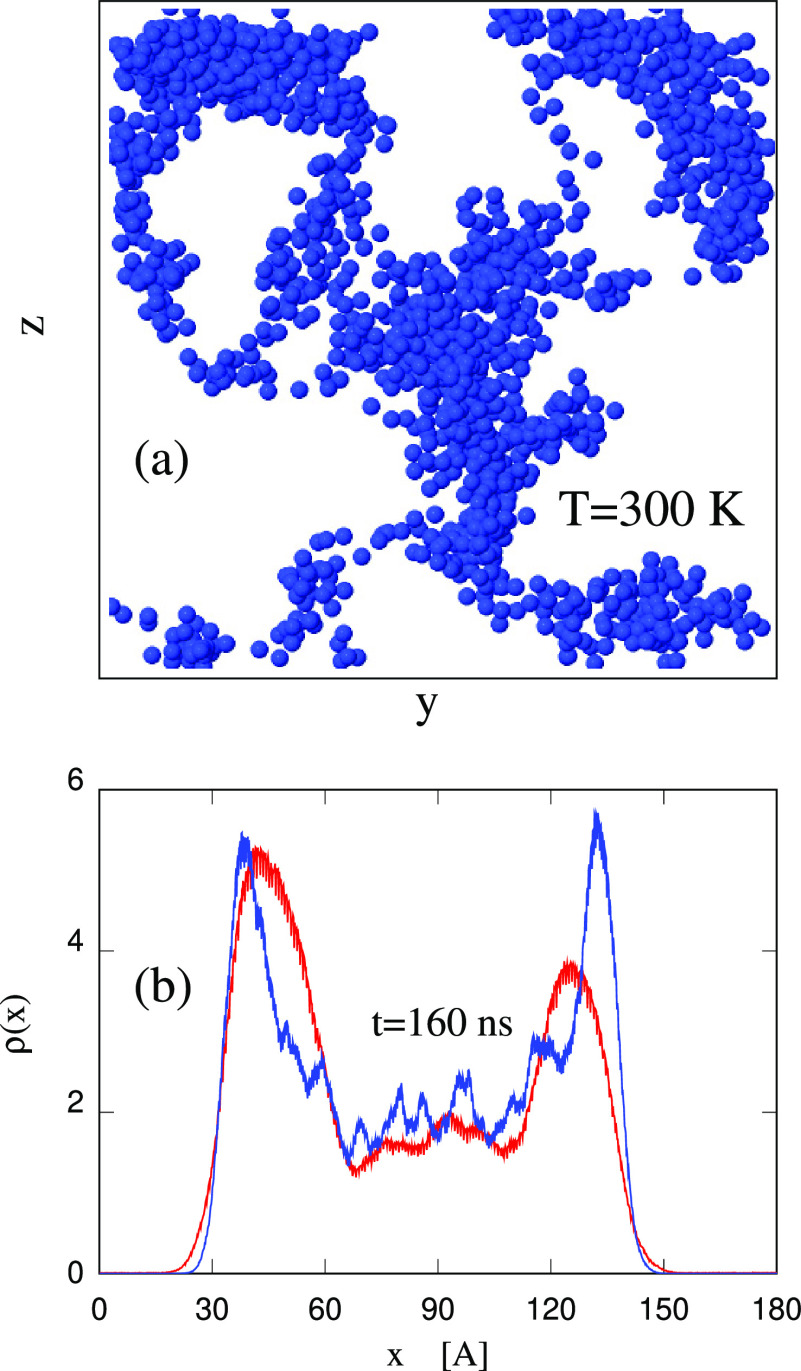
(a) Largest 2-connected cluster of water molecules in the sample consisting of the (90–10) wt % slab on which 2800 water molecules have been deposited. The cluster is responsible for one of the two subsurface peaks in the water density profile at *t* = 160 ns shown in (b). The cluster in this figure consists of 1754 water molecules and is seen from the directional orthogonal to the slab. (b) Temperature dependence of the water density profile for the (90–10) wt % slab on which 2800 water molecules have been deposited. Blue line: *T* = 300 K; red line: *T* = 360 K. Part (a) and (b) are not on scale. The sides of the upper square are *L*_*y*_ = *L*_*z*_ = 10.85 nm.

The real-space nanostructuring analysis shows that the subsurface peaks in the water distribution reflect the formation of large clusters made predominantly but not exclusively of water. At 300 K, for instance, of the total 7120 molecules in the sample, about 2 × 1700 form two well-defined clusters corresponding to the two subsurface peaks. As expected, the connectivity index *p* of water molecules in the clusters (see Section II) increases with increasing water content, with the 2-connectivity following closely the 1-connectivity, while the 3-connectivity (see Section II) still covers a relatively small faction of all water molecules. Visualization of nanostructuring shows that water gives origin to sizable water-rich domain joined by thick filamentous threads, as apparent in Figure 8, showing the largest subsurface 2-connected cluster found in a randomly chosen snapshot at *T* = 300 K. On this size scale, nanostructuring could already ease the separation by mechanical means of water-rich and IL-rich phases even before the macroscopic demixing transition.

The water subsurface peaks become broader with increasing temperature, as shown in Figure 8. The broadening, however, does not correspond to a uniform spreading of water across the system. Comparison of the water density profiles at *T* = 360 K and at *T* = 300 K at equal *t* = 160 ns even suggests that, at the highest temperature, the two water-rich subsurface domains are able to pull water from the inside, consistent with the fact that nanostructuring, i.e., limited water-IL separation, becomes stronger with increasing *T*. Furthermore, comparison of the clustering data confirms that water clusters are larger and more connected at *T* = 360 K than at *T* = 300 K. The broadening of the peaks, therefore, is due to a change in the position and shape of the water-rich domains that become more globular and move slightly deeper into the slab without losing their connectivity.

#### 3 Simulations of Slab Samples with up to 25 wt % Water Concentration

Further simulations have been carried out over a broad temperature range for samples with significantly more water to probe the kinetics of large subsurface clusters and to investigate whether strong surface clustering is affected by higher water content. At 25 wt % water in [P_4444_][DMBS], no thermodynamic demixing occurs over the liquid water range of temperatures, but the system properties are greatly affected by nanostructuring especially at the highest temperatures. The persistence of water-rich domains up to long time scales is reflected in the fact that increasing the number of water molecules in the sample increases mainly the number of water–water HBs (from 0.80 to 1.20 HBs donated/accepted per water molecule), while the increase of water–anion HBs is much less than proportional, growing from 1.75 in the (90–10) % slab to 1.82 in the 25 wt % water slab.

**Figure 9:**
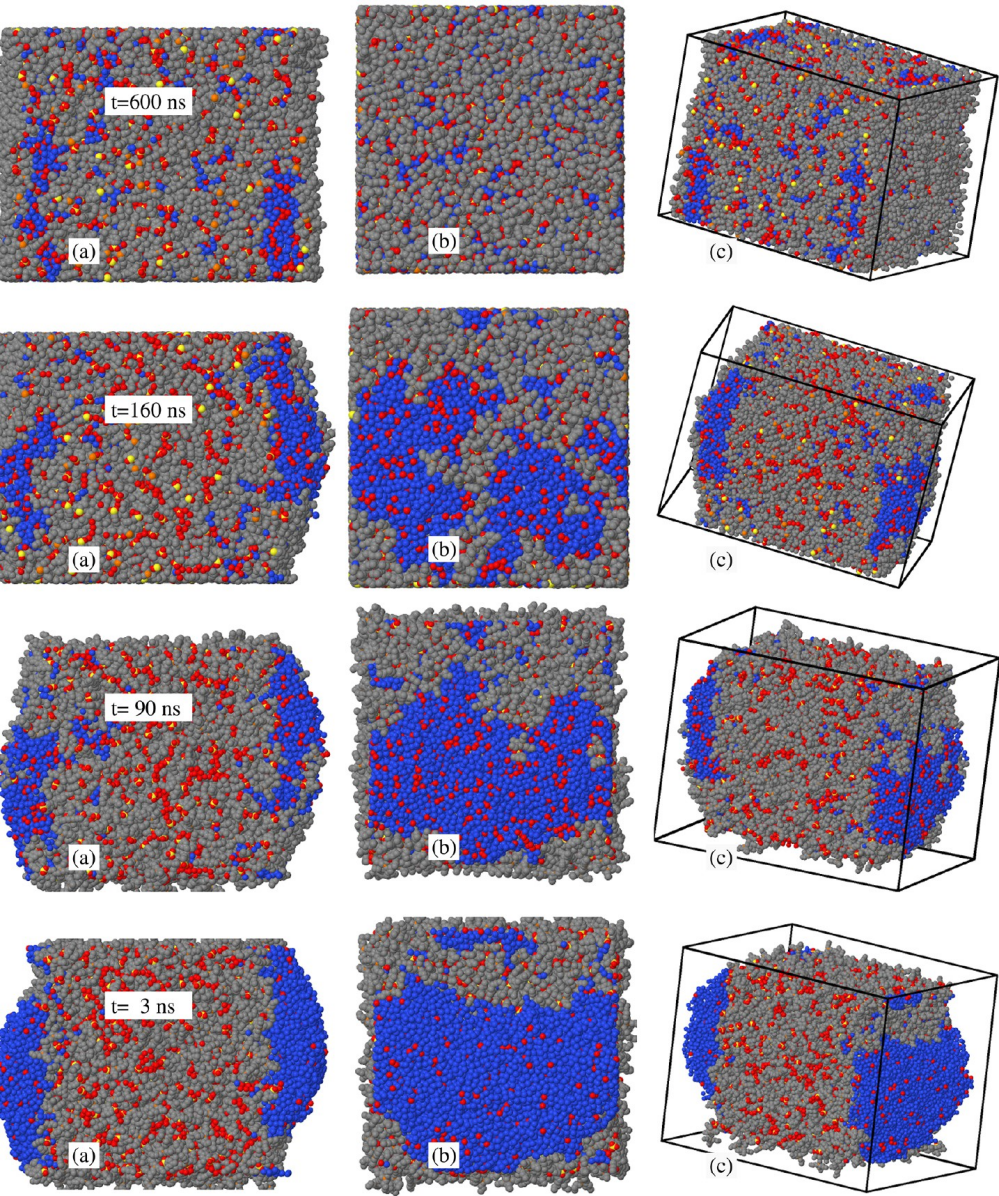
Time evolution of the sample consisting of the (90–10) wt % slab at *T* = 300 K to which 8900 water molecules have been added according to the procedure described in the text. Column (a) provides a side view of the slab, with the two exposed surfaces on the left and right sides of the picture. Column (b) is a top view of the left surface in Column (a). Column (c) is a tilted view, intended to ease the connection between views (a) and (b). The rapid deposition of water forms large 3D mounds on the (90–10) wt % slab surfaces. With increasing time, ions migrate above the exposed water surface, lowering the sample surface free energy. The process is completed in about 500 ns. Gray dots: C atoms; red dots: oxygen belonging to [DMBS]^−^ and to water molecules already present in the sample; blue dots: oxygen of the added water molecules; orange dots: P atoms; yellow dots: S in [DMBS]^−^. H is not shown.

First, we simulated samples consisting of the (90–10) wt % slab upon the addition of 8900 water molecules (∼2.5 ML). As before, the addition has been carried out in stages, each time adding 1000 molecules and briefly equilibrating the sample before the following addition. Considering the water already present in the slab, the system contains 13 220 water molecules. At *T* = 300 K simulations cover 700 ns, and a further simulation for the same sample at *T* = 320 K has been extended to 1.6 μs. The time evolution of the sample following the water addition is documented in Figure 9 for *T* = 300 K. At first (3 ns row), water forms large 3D dune-like mounds on the surface, also in this case sitting in a concavity formed by bending inward the IL surface. Mobility of native and added water is good, and the two water populations tend to mix already in the first stages of the system evolution, again primarily by drawing water from the inside to the water islands on the surface. Then, on the time scale of 100 ns (90 ns row), both cations and anions start to climb the side of the water mounds. About 150 ns after water deposition (160 ns row), the process is well under way, but the coverage is not uniform, with exposed water islands and ribbon-like ion structures extending on the water surface. The process of burying water under an IL film is virtually completed after half μs (600 ns row). This sequence of observations suggests that the mechanism of ions covering the surface of adsorbed water droplets is potentially able to incorporate large amounts of water into the slab within a microscopic time, which increases with increasing lateral size of the islands but depends only weakly on their thickness.

**Figure 10:**
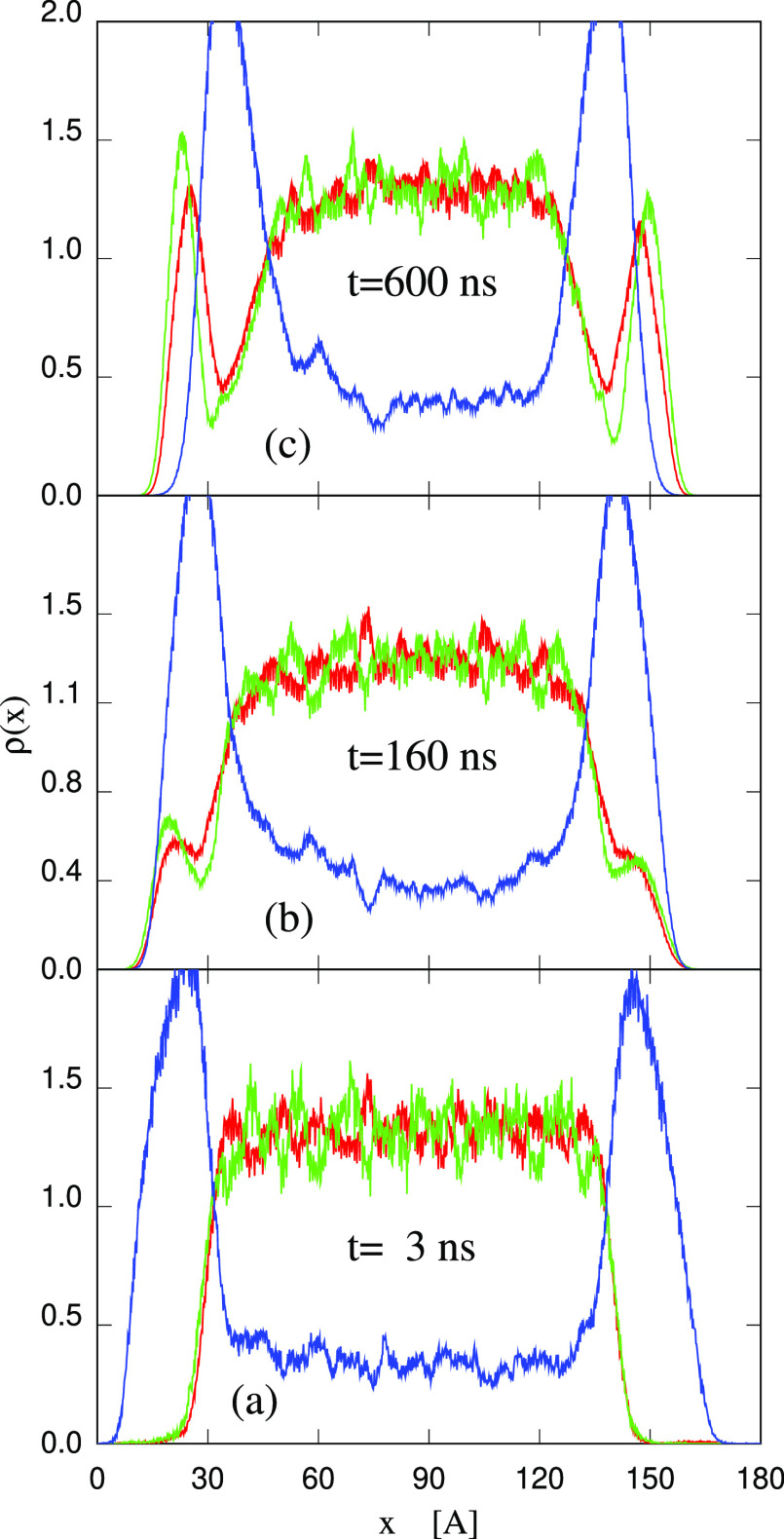
Time evolution of the ion and water density profiles following the random addition of 8900 water molecules in the empty space in between periodically repeated (90–10) wt % slabs at *T* = 300 K. Therefore, the sample consists of 1728 [P_4444_][DMBS] ion pairs and 13 220 water molecules. Red line: [P_4444_]^+^; green line: [DMBS]^−^; blue line: H_2_O.

The evolution illustrated in Figure 9 is reflected in the corresponding changes in the water density profile shown in Figure 10. Shortly after the water deposition and brief equilibration between successive additions of 1000 water molecules, the water density distribution shows an approximately constant density inside the slab, corresponding to the original 10 wt % water content, and two high and relatively broad peaks corresponding to the 3D water mounds on the surface. On the 100 ns time frame, the cation and anion density distributions develop shoulders on their sides, corresponding to the ions that crawl on the water-free surface. Over a longer time scale (∼0.5 μs), the shoulders become sizable peaks, confining water below the surface. In the bulk at these same concentrations, the diffusion constant of water is already half of the pure water value, and the cation and anion diffusion constants, although 2 orders of magnitude smaller, are already significant. The relative stability of the water domains just below the surface, therefore, is a combination of kinetics and thermodynamic factors, related to the fact that nanostructuring leaves only a small free energy margin to drive the dissolution of sizable water pockets and their penetration toward the inside of the slab.

**Figure 11:**
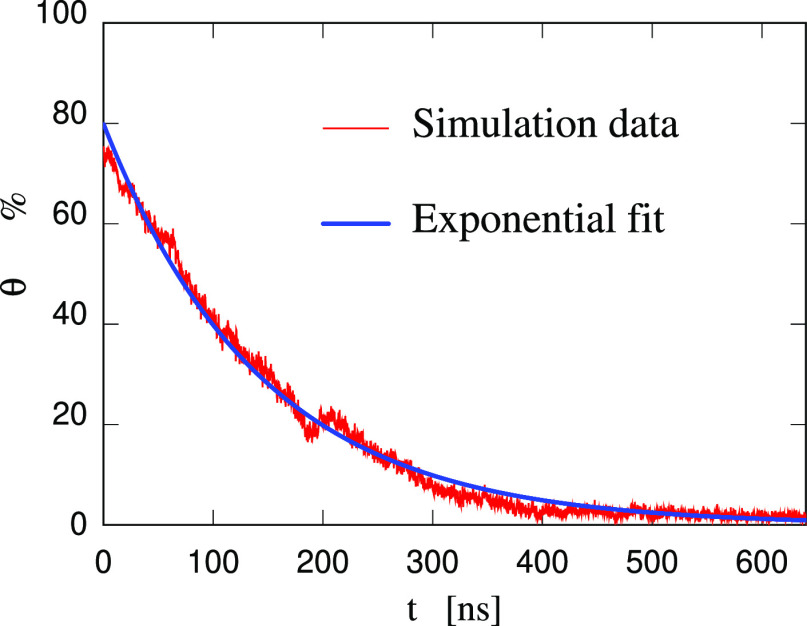
Time evolution of fraction θ (in %) of the surface area covered by water. Same sample, temperature *T* = 300 K, and time scale of Figure 9.

This qualitative description of the absorption process has been made quantitative by computing the time evolution of the fraction θ (in %) of the surface covered by water. To this aim, the *v*AFM approach of ref (41) (briefly mentioned in Supporting Information) has been used on a regular sequence of snapshots separated by 0.2 ns along the simulation trajectory at *T* = 300 K (see Figure 11). Shortly after deposition, as already seen in Figure 9a, each of the two surfaces of the (90–10) wt % slab is 80% covered (θ(3 ns) = 80) by a thick water drop. With increasing time, θ(*t*) decreases steadily, following an approximate exponential law of time constant τ = 145 ns. At long time, θ(*t*) fluctuates around 1%, corresponding to the occasional exposition of water molecules from the slab to the outside.

Increasing temperature increases at the same time the mobility of all species as well as the tendency to produce domains of larger size, which, in turn, decreases the overall mobility of the domains. Thus, the effect of temperature on the evolution of mesoscopic features is only weakly affected.

Since no genuine phase transition is detected in the system, the results of simulations carried out at high temperature (up to *T* = 360 K) provide insight on the system evolution over slightly longer (multi-μs) time scales at lower *T*. Comparison of the water density profiles computed at comparable times shows that temperature does not change the results qualitatively up to *T* = 360 K (see Figure S8 in Supporting Information).

**Figure 12:**
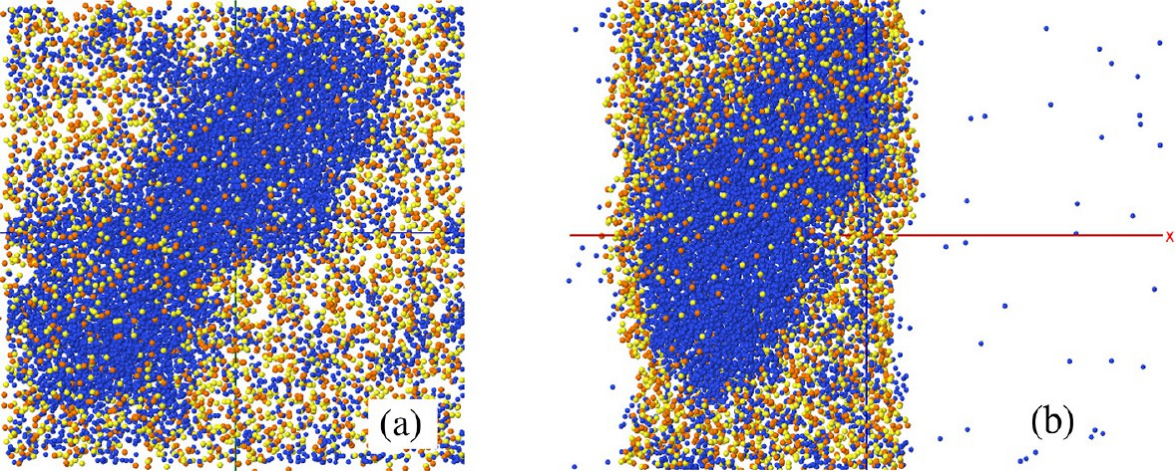
Top (panel (a)) and side (panel (b)) views of the IL slab with *N*_w_ = 12 000 absorbed water molecules at *t* = 500 ns after water deposition. Water molecules are represented by the blue dots. To increase the visibility of the large water-rich domain, the C and O atoms belonging to the ions have been removed, and all H atoms have not been represented.

In a last series of simulations, we investigated the addition of 12 000 water molecules on the dry [P_4444_][DMBS] surface, whose final composition is similar to the one of the (90–10) wt % slab plus the 8900 water molecules. As expected, the results are qualitatively similar to those discussed for the (90–10) wt % slab +8900 water molecules. Only the long-time kinetics, investigated by increasing temperature, is somewhat different because of the slow penetration of water deep inside the dry [P_4444_][DMBS] salt. For instance, at *T* = 360 K the subsurface peaks evolve into a structureless density distribution filling the inside of the [P_4444_][DMBS] slab, leaving a thin [P_4444_][DMBS] topmost layer exposed on the two surfaces. Once again, the smoothing of the density profile is not due to the dissolution of water-rich domains but rather to their change of shape, becoming more globular, and to their migration toward the center of the slab as shown in Figure 12.

In general, we think that the long-term fate of the sample morphology is determined by the equilibrium bulk phase diagram presented in ref (25). Nevertheless, the subsurface absorption pockets are likely to play a role in the system kinetics at high absorption rates. The good news is that the system quickly renews its topmost layer, which, although hydrophobic, favors the incorporation of water within the slab. The bad news is that the strong tendency toward water nanostructuring in [P_4444_][DMBS]/water solutions might represent a kinetic bottleneck that prevents the rapid diffusion of water molecules toward the bulk, since water-rich aggregates are relatively stable and difficult to break. In view of water-harvesting applications, however, this last problem could be eased, for instance, by continuously stirring the IL/water solution while exposed to the atmospheric vapor.

## IV Summary and Conclusions

Harvesting the ubiquitous and globally abundant but thinly spread water vapor dissolved in the atmosphere could represent a valuable source of water in vast arid and semiarid regions. Thermoresponsive materials could help achieve this goal at a lower energy cost, breaking the so-called water-energy connections. More precisely, they could make the energy requirement more affordable by using inexpensive heat from the environment, exploiting daily temperature oscillation. In this respect, thermoresponsive ionic liquids presenting a so-called lower critical solution temperature (LCST) demixing could absorb water vapor during the night, releasing liquid water during the day. While the basic mechanism underlying this approach relies on mixing/demixing bulk equilibrium properties that are fairly well-known, the real-life feasibility of water harvesting through thermoresponsive ILs depends on several other less well-known issues.

In our study we address two such issues, related to the kinetics of water absorption at the IL surface and to the role of nanostructuring of IL/water solutions. To investigate these aspects, atomistic MD simulations have been carried out for systems made of the [P_4444_][DMBS] ionic liquid, whose water solutions present an LCST at *T*_c_ = 309 K and *x*_c_ = 50–50 wt % composition.^25^ A wide temperature range has been explored, corresponding to 260 ≤ *T*, K ≤ 360, with the lowest and highest temperatures justified by the fact that the IL-water solution has a broader liquid range than simply water, and the extreme temperatures help to amplify kinetic effects. At least for *T* ≥ 280 K, [P_4444_][DMBS] is liquid-like, a necessary condition for any potential application in water harvesting. The system fluidity is systematically and significantly increased by the addition of water, whose mobility in [P_4444_][DMBS] is about 2 orders of magnitude higher than that of the ions.

The kinetics of absorption has been probed starting from either (i) a dry slab or (ii) a water-contaminated slab whose relative composition in IL and water was (90–10) wt %. This last composition was selected because it corresponds to that of the IL-rich phase produced by demixing at the LCST point. On these starting slabs, water has been deposited up to ∼3 ML coverage (each side), representing ∼25 wt % of the sample. Water coverage did not reach the optimal 50–50 wt % composition because, at the expected absorption rate of ∼1 mm per day and a practical IL sorbent thickness of a few millimeters, it seems unlikely to reach such a high water content during one night in an arid environment.

The results show that both sorbent slabs present free surfaces whose outermost layer is predominantly populated by the a-polar side chains of either cations and anions. Despite the hydrophobic nature of the surface, water sticks to the slab mainly because of dispersion forces, at first forming randomly distributed physisorbed clusters. These initial aggregates coalesce into larger, thick 3D adsorbed domains, as expected for water on hydrophobic surfaces. On the 10^2^ ns time scale, water islands begin to be buried below the surface. Surprisingly, this process is not due to a displacement of the mobile water but of the viscous IL, whose ions crawl on the water domains to isolate them from the vacuum phase, thus quickly regenerating the nearly pristine IL surface. The process is driven by the low surface tension of the amphiphilic ions, whose a-polar termination has low surface energy and gains much entropy with increasing temperature. The immediate result is the accumulation of water in subsurface domains, at the beginning rather well confined in the direction perpendicular to the surface. Following the absorption stage, the evolution of the water domains is very slow, reflecting the low free energy of the nanostructured configurations. In all cases, over the longest time scales of our simulations, water-rich domains do not dissolve into the IL. In fact, they grow slowly, drawing water from the inside of the water-contaminated slab.

The simultaneous burying of entire water domains by the migration of the ions is a new absorption mechanism not previously discussed in the literature. The same collective absorption mechanism is observed in all of our simulations, starting from either the pure or the water-contaminated sorbing slab. Temperature and coverage affect the kinetics and details of the IL/water nanostructuring during and immediately following the water absorption.

All together, these observations suggest that the kinetics of water absorption in a widely investigated thermoresponsive IL is compatible with its application in water harvesting from the atmosphere. Following absorption, IL/water nanostructuring might slow down the progression of water domains toward the center of the slab, needed to turn absorption from a surface process to a volume process. However, the problem might be cured by stirring the solution. On the other hand, the mechanism of covering water islands by ions migrating on their surface allows the fast incorporation in the IL of large amounts of water, compensating the disadvantage in surface area with respect to porous solids and bringing within reach the ∼1 kg/day/m^2^ specific water production of competing methods. Moreover, and perhaps more importantly, coarse nanostructuring could allow the separation of the IL-rich and water-rich phases even below the temperature of macroscopic demixing using simple mechanical means such as sedimentation under gravity or centrifugation. According to the present and previous studies,^25^ the IL-rich phase obtained in this way contains about 10 wt % water, whose presence, according to our results, has only a weak effect on the further absorption process. Therefore, the IL-rich phase regenerated by the separation process can be used as-is in a new cycle. The water-rich phase contains a low concentration of [P_4444_]^+^ and [DMBS]^−^ ions that is difficult to measure quantitatively using the small, inhomogeneous samples of the present simulation. It is likely, however, that this phase needs to be further purified to be used in agriculture or for human consumption. Moreover, further purification might be needed for the complete recovery of the expensive IL. However, the osmotic pressure of the water-rich solution is certainly low (since the density of ions appears to be low), and the final separation, for instance, by nanofiltration, could be simple and inexpensive.

Our discussion, focused on scientific and technical aspects of feasibility, does not touch upon other important factors, such as the cost of a massive device based on ILs, or the chemical stability of the crucial IL component while dissolving water and a variety of contaminants. These aspects might indeed represent the strictest requirements, limiting the practical relevance of the process. In this respect, ILs are known for their low dispersion in the environment by evaporation, favoring a very high degree of IL recycling and conservation. Moreover, to some extent, the price depends on the scale of production and on incremental improvements that would be stimulated by extensive usage.

## Supporting Information Available

A short theory section describing the computation of the surface tension γ and a series of figures on structural, thermodynamic and dynamic properties of [P_4444_][DMBS]/water solutions, including average potential energy as a function of temperature; mean square displacement as a function of time of ions and water; density profiles across slab samples; characterization of fluctuations in the surface position. (PDF)
