# Supplementary material for: Water Harvesting by Thermoresponsive Ionic Liquids: A Molecular Dynamics Study of the Water Absorption Kinetics and of the Role of Nanostructuring
Source: J Phys Chem B. 2023 Jun 2;127(24):5494–508. doi: 10.1021/acs.jpcb.3c01655 (PMC10291554; doi:10.1021/acs.jpcb.3c01655)
Supplement: Supplementary file 1 — jp3c01655_si_001.pdf [file jp3c01655_si_001.pdf]

Supporting Information on:

Water Harvesting by Thermoresponsive Ionic Liquids: A Molecular Dynamics Study of the Water Absorption Kinetics and of the Role of Nanostructuring

Nancy C. Forero-Martinez,[1,2] Robinson Cortes-Huerto,[2,*] Lainey Ward,[3] and Pietro Ballone,[3,4]

(1) Institut für Physik, Johannes Gutenberg-Universität Mainz, Staudingerweg 9, 55128 Mainz, Germany

(2) Max-Planck Institute for Polymer Research, Ackermannweg 10, 55128 Mainz, Germany

(3) School of Physics, University College Dublin, UCD Belfield Campus, Dublin 4, Ireland

(4) Conway Institute for Biomolecular and Biomedical Research, University College Dublin, UCD Belfield Campus, Dublin 4, Ireland

I. COMPUTATION OF THE SURFACE TENSION γ FROM THE EQUILIBRIUM FLUCTUATIONS OF THE SURFACE

Under widely verified conditions, and taking the average planar surface (orthogonal to $\hat{\mathbf{x}}$) as a reference, the instantaneous surface position $x(y,z)$ is written as the superposition of plane wave undulations. Because of the periodic boundary conditions employed in the simulation, also the $x(y,z)$ function is periodic along the surface, and can be written as:

$$x(y,z) = \sum_{\mathbf{q}\in\Omega} C_{\mathbf{q}} e^{i\mathbf{q}\mathbf{r}_{yz}} \tag{1}$$

where Ω is the set of discrete reciprocal lattice vectors $\{\mathbf{q}\}$ of the periodic surface, and $\mathbf{r}_{yz}$ is the projection of $\mathbf{r}$ on the underlying planar surface. Then, a relation widely used for liquid surfaces states that in the limit of long wavelengths (low $|\mathbf{q}|$):[1S,2S]

$$\langle C_{\mathbf{q}} C_{-\mathbf{q}} \rangle = \frac{k_B T}{A_0} \frac{1}{\gamma q^2 + K_c q^4} \tag{2}$$

where $\langle ... \rangle$ indicates average over time, A_0 is the projection of the surface the xy plane, and K_c is the surface bending rigidity. As explained above, the orthorhombic shape of the simulation box has been selected in such a way that the surface is orthogonal to the x axis, and $A_0 = L_p \times L_p$. Needless to say, to apply this method one needs first to determine the position $x(y,z)$ of the

two parallel surfaces delimiting the slab. This can be done using a method that we could call virtual atomic force microscopy *vAFM*, already used in previous computations, and detailed, for instance in Ref. 3S. The method is fast, reliable, and produces maps of the surface on a regular grid that can be efficiently Fourier transformed. One drawback is that, once again, molecules belonging to the dilute water vapour could spoil the determination of the position x for a few (y, z) on the surface. To prevent this problem, all water molecules whose coordination by non-hydrogen atoms is ≤ 2 are removed from the configuration before the surface is analysed. This empirical approach seems to work, but it also introduces a (small) degree of arbitrariness.

This approach to compute γ (and, of course, K_c) is viable as far as the amplitudes of the fluctuations can be effectively averaged during the limited time of the simulation. With decreasing $|\mathbf{q}|$, however, the period of the wave-like excitations increases ($\omega \propto q^{3/2}$ for capillary waves), making sampling more challenging or at least more time consuming. The problem is particularly severe for viscous systems like pure $[P_{4444}]$[DMBS], but the addition of water makes the task somewhat easier. In all cases, the problem can be solved by neglecting one or two shells of $\mathbf{q}$ vectors of lowest modulus $|\mathbf{q}|$.

[1S] Waheed, Q.; Edholm, O. Undulation contributions to the area compressibility in lipid bilayer simulations. *Biophys. J.* **2009**, *97*, 2754-2760.

[2S] Lindhal, E.; Edholm, O. Mesoscopic undulations and thickness fluctuations in lipid bilayers from molecular dynamics simulations. *Biophys. J.* **2000**, *79*, 426-433.

[3S] Pillai, V. V. S.; Kumari, P.; Benedetto, A.; Gobbo, D.; Ballone, P. Absorption of phosphonium cations and dications into a hydrated POPC phospholipid bilayer: A computational study. *J. Phys. Chem. B* **2022**, *126*, 4272-4288.

II. MISCELLANEOUS FIGURES

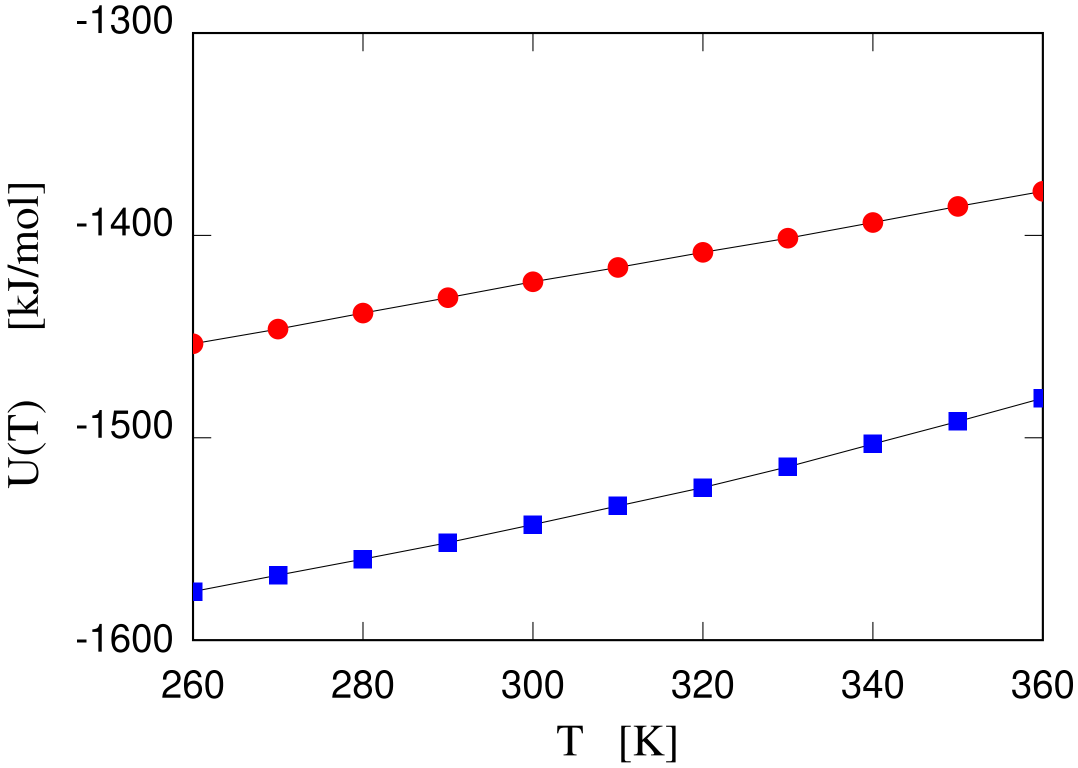

FIG. S1: Average potential energy as a function of temperature of homogeneous samples. Red dots: dry sample. Blue squares: (90-10) wt% sample. Each sample contains 1728 neutral ion pairs ($[P_{4444}]^+$; $[DMBS]^-$). The (90-10) wt% sample contains also 4320 water molecules. More precisely, $U(T)$ is the average potential energy of the whole sample divided by the number of ion pairs.

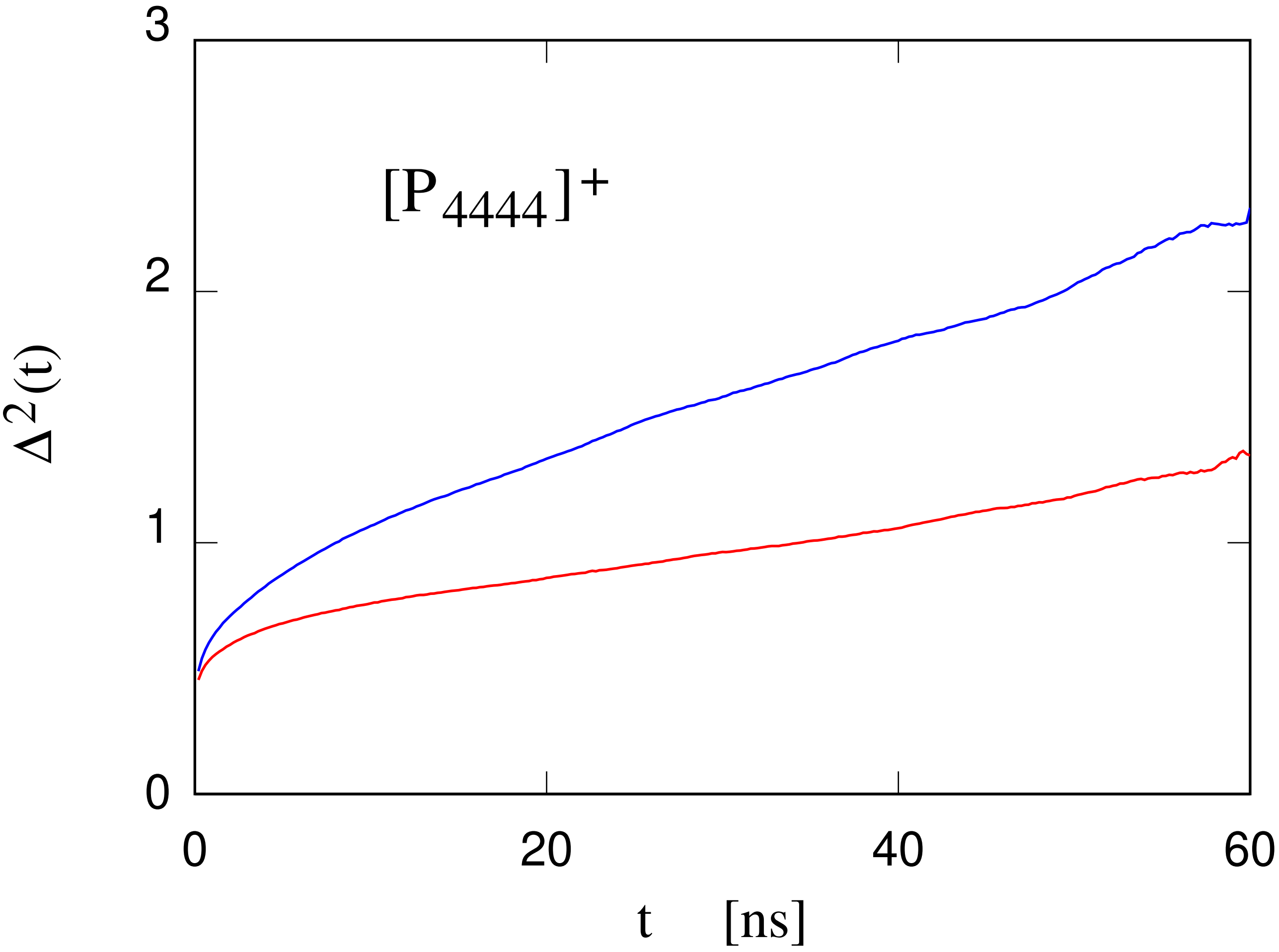

FIG. S2: Comparison of the mean square displacement as a function of time of the $[P_{4444}]^+$ ions in two homogeneous samples at $T = 300$ K and $P = 1$ bar. Red line: dry $[P_{4444}]$[DMBS] sample; blue line: wet $[P_{4444}]$[DMBS] of IL-water composition (90-10) wt%, respectively. Linear interpolation on the time interval $40 \leq t \leq 50$ ns gives an estimate for the $[P_{4444}]^+$ diffusion constant of $D = 2.1 \pm 0.3 \times 10^{-8}$ cm^2/s in the dry sample, and $D = 3.7 \pm 0.3 \times 10^{-8}$ cm^2/s in the (90-10) wt% sample. In both samples, the diffusion constant of the anion is slightly lower than the one for the cation.

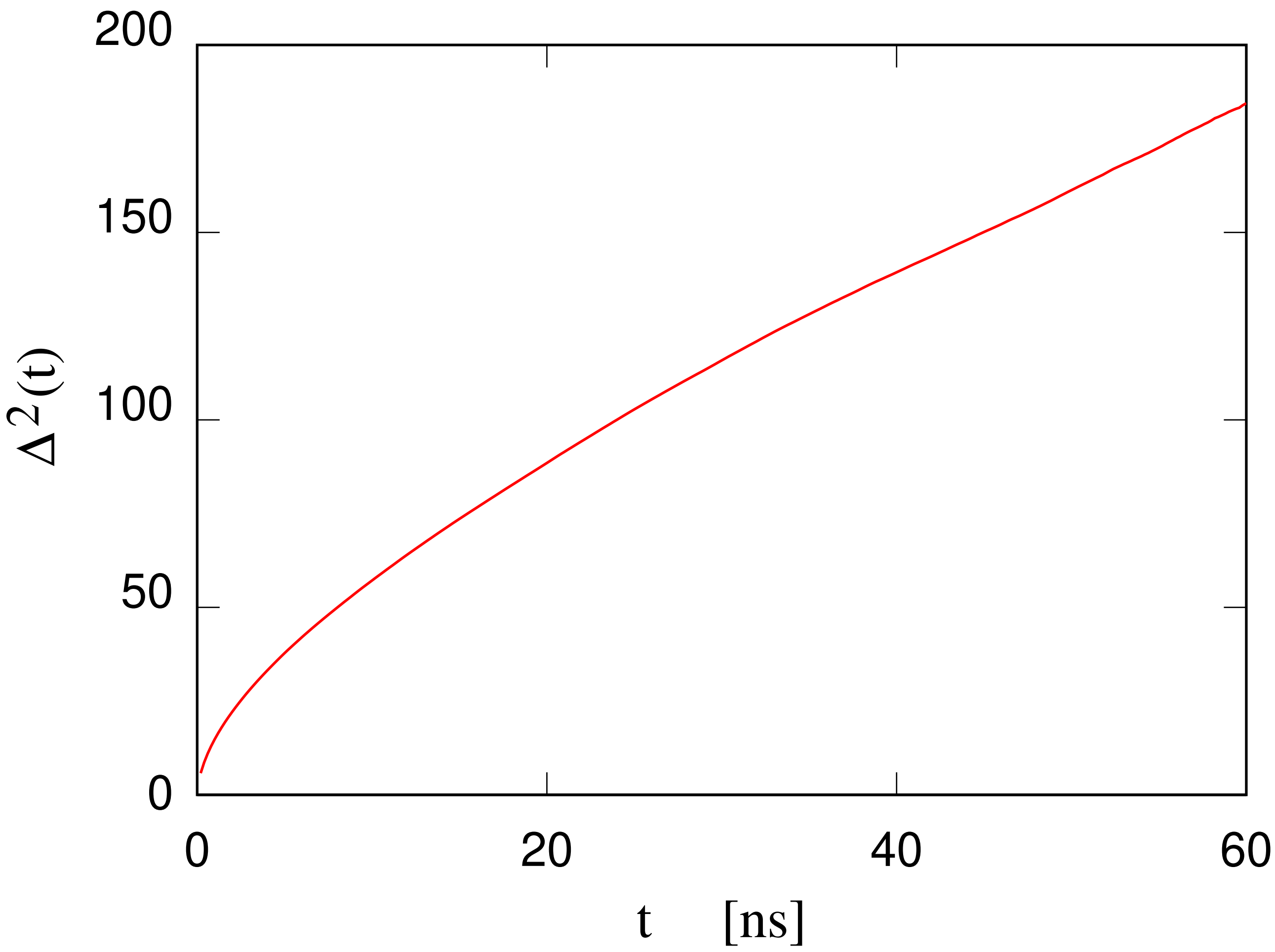

FIG. S3: Mean square displacement as a function of time of water molecules in the (90-10) wt% homogeneous system at $T = 300$ K and $P = 1$ bar. Linear interpolation on the interval for $40 \leq t \leq 50$ ns gives an estimate of $D = 3.65 \pm 0.1 \times 10^{-6}$ cm^2/s.

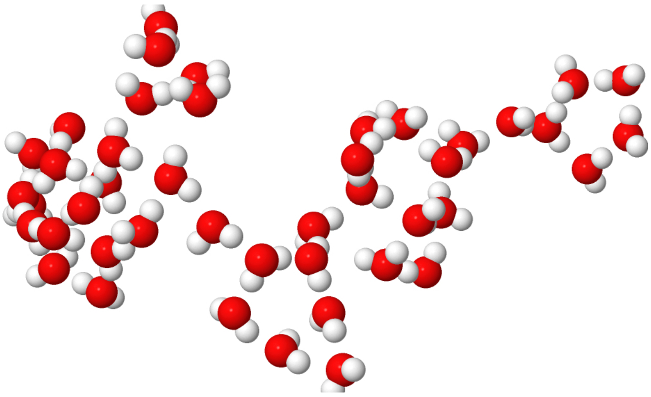

FIG. S4: Largest 2-connected water cluster found in a random snapshot from the simulation of the (90-10) wt% homogeneous system at $T = 300$ K and $P = 1$ bar.

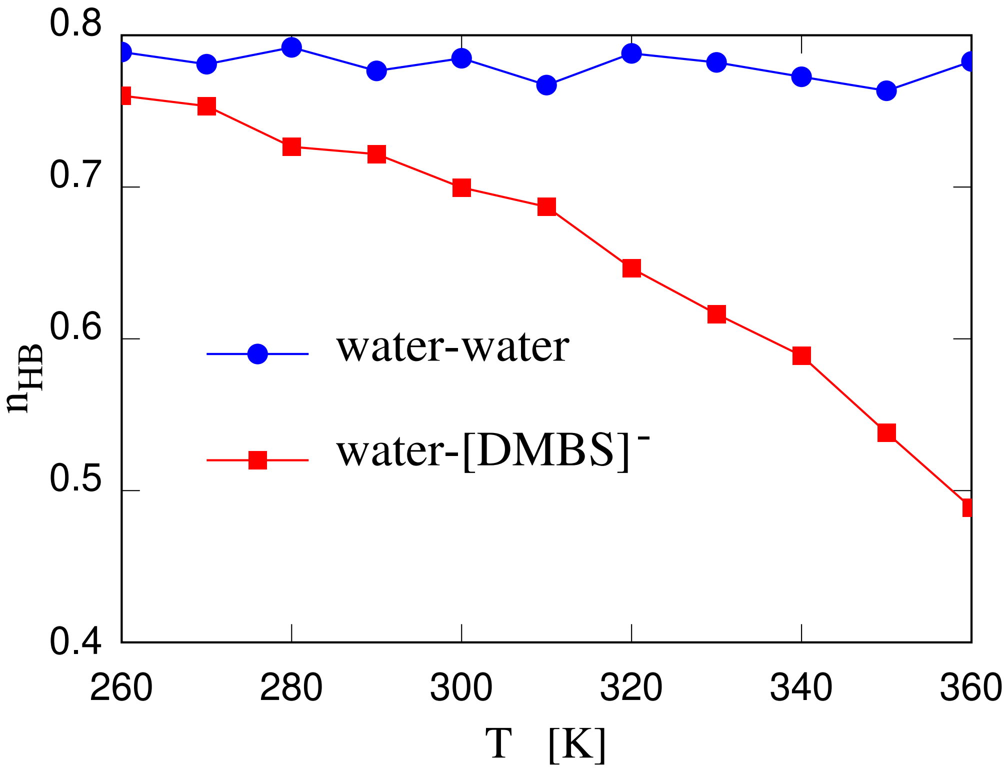

FIG. S5: Average number n_{HB} of hydrogen bonds donated by each water molecules to: water molecules (blue line and dots); $[DMBS]^-$ anions (red line and dots). The continuous lines are a guide to the eye.

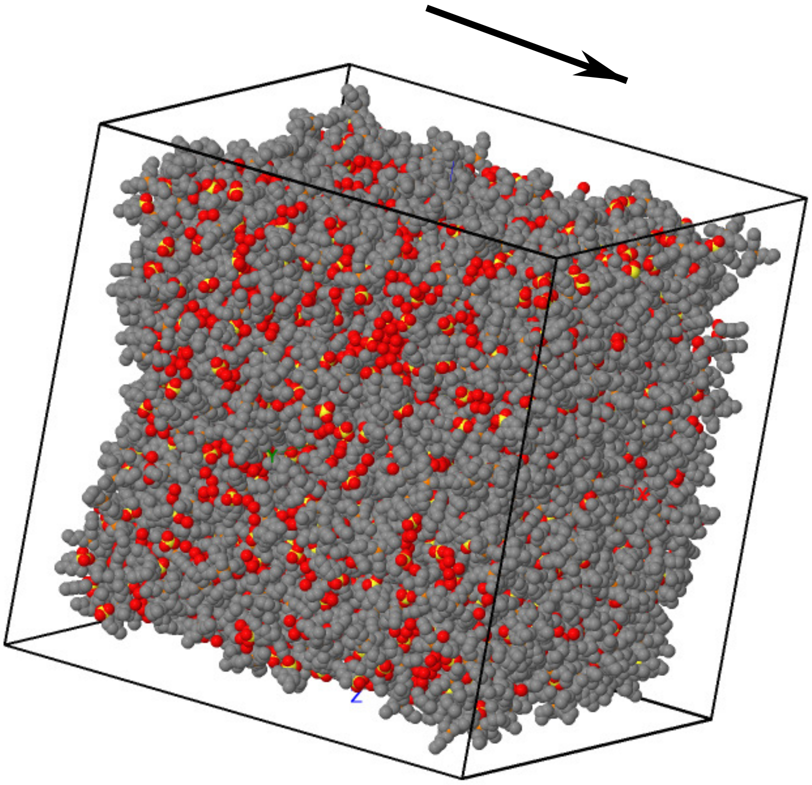

FIG. S6: Snapshot from the simulation of the (90-10) wt% slab at $T = 300$ K. The arrow on top show the direction normal to the surface. The two surfaces exposed to the vapour side are predominantly populated by the hydrocarbon tails of $[P_{4444}]^+$ and $[DMBS]^-$.

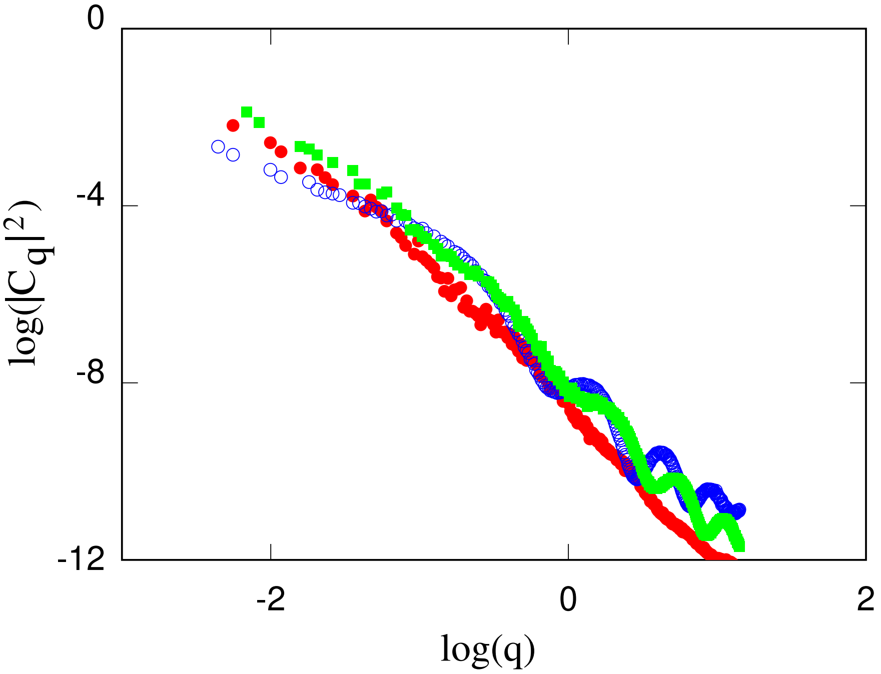

FIG. S7: Equilibrium fluctuations of the surface of: water (blue circles); [P_{4444}][DMBS] (red dots); and [P_{4444}][DMBS] / water solution at 10 wt% water composition (green dots). The C_q coefficients are computed according to the approach described in the text. Fit of the related $h(q) = \langle |C_q|^2 \rangle$ with the function $h(q) = (k_B T/A_0)/(\gamma q^2 + k_c q^4)$ allows to estimate the surface tension γ and the bending rigidity K_c. In these expressions, $\mathbf{q}$ are 2D reciprocal lattice vectors of the simulation box along the (y, z) plane of the surface, A_0 is the average surface area, k_B is the Boltzmann constant, and T the sample temperature. The surface tension of water is estimated at 48 mJ/m^2, in line with literature value for SPC water, and underestimated with respect to the experimental value. The ratio of surface tension γ in water, [P_{4444}][DMBS] and [P_{4444}][DMBS] / water solution is 1:0.37:0.37. The error bar on the surface tension is of the order of 10%. The accuracy of the computation is not sufficient to estimate the bending rigidity with an acceptable error bar.

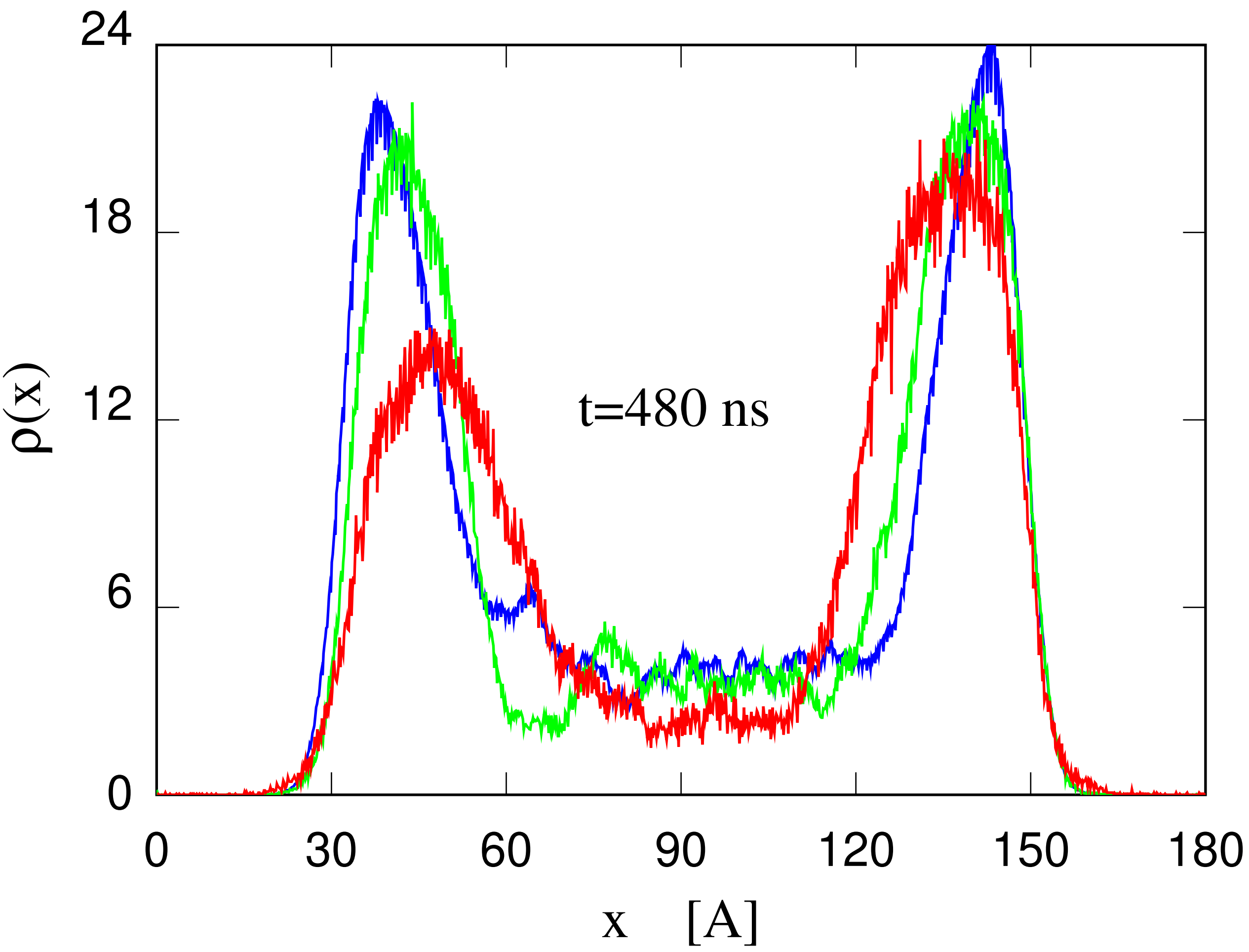

FIG. S8: Temperature dependence of the water density profile for the (90-10) wt% slab on which 8900 water molecules have been deposited. Blue line: $T = 300$ K; green line: $T = 320$ K; red line: $T = 360$ K. Despite the relatively long time and the sizeable temperature difference the water profiles are still relatively similar, showing that the increasing tendency to nanostructuring prevents the dissolution of large water islands after their incorporation into a subsurface layer. Even the broadening of the peaks at $T = 360$ K is not due to the system becoming homogeneous, but to the variation in the orientation and position of two large connected domains located below the surface. Again at $T = 360$ K, it is apparent that the two water-rich domains are able to draw water from the middle of the slab, as shown by the lower water density at the centre.
